# Quantitative Analysis of DNA Double‐Strand Breaks in Genomic DNA Using Standard Curve Method

**DOI:** 10.1002/jcla.70123

**Published:** 2025-10-18

**Authors:** Lihuang Guo, Hanying Dai, Jiancheng Li, Chenwei Li, Yue Huang, Keqian Xu

**Affiliations:** ^1^ Department of Laboratory Medicine, the Third Xiangya Hospital Central South University Changsha China; ^2^ Department of Laboratory Medicine, Xiangya School of Medicine Central South University Changsha China; ^3^ Department of Clinical Laboratory Shenzhen Traditional Chinese Medicine Hospital Shenzhen China

**Keywords:** DNA damage, DNA double‐strand breaks, ligation‐mediated real‐time quantitative PCR, standard curve method

## Abstract

**Background:**

DNA double‐strand breaks (DSBs) are the most lethal and dangerous type of lesions with significant implications for both cellular function and organismal health. The number of DSBs (N_DSBs_) across the genome reflects DNA damage severity. However, current quantification methods mainly rely on next‐generation sequencing, which is laborious and expensive. This study aims to provide a simple, low‐cost, and high‐throughput standard curve‐based method for quantifying genome‐wide DSBs.

**Method:**

Genomic DNA from human, mouse, *Arabidopsis*, *Saccharomyces cerevisiae* , and *Escherichia coli* was digested by seven blunt‐end restriction enzymes to generate DSB standards. Theoretical N_DSBs_ for each standard were calculated based on restriction site frequency. Ligation‐mediated quantitative PCR (LM‐qPCR) was performed to obtain the Ct values, which were plotted against log‐transformed N_DSBs_ to construct standard curves. Method reliability was assessed by comparing results with neutral single‐cell gel electrophoresis and γ‐H2AX flow cytometry.

**Results:**

All genomes were successfully digested by seven blunt‐end restriction enzymes to produce standard DSB fragments. Standard curves demonstrated high linearity (*R* ^2^ > 0.95), with intra‐ and inter‐assay coefficients of variation of 1.101% and 2.528%, respectively. The detection limit was below 100 DSBs. Quantification results strongly correlated with traditional DSB detection methods (|*r*| > 0.9).

**Conclusion:**

This standard curve‐based method enables accurate, reproducible quantification of genome‐wide DSBs in various organisms. It is simple, low‐cost, and easily standardized, offering a promising tool for applications in genotoxicity testing, environmental exposure monitoring, and DNA damage research.

## Graphical abstract

This study presents a simple and cost‐effective method for quantifying DNA double‐strand breaks (DSBs) using standard curves based on Ct values from LM‐qPCR and the theoretical number of DSBs from enzyme‐digested DNA standards. Its simplicity, affordability, and high‐throughput capability make it a valuable tool for genotoxicity testing, environmental monitoring, and DNA damage studies across diverse organisms.

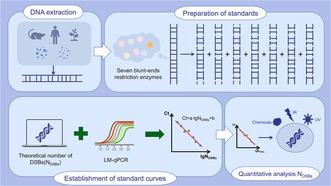

## 1 Introduction

Genomic DNA is constantly exposed to a variety of endogenous and exogenous factors, including environmental agents and pollutants, which can cause varying degrees of DNA damage, such as base modifications, DNA single‐strand breaks (SSBs), and DNA double‐strand breaks (DSBs) [1]. It has been estimated that each mammalian cell undergoes approximately 200 cytosine deaminations, 3000 guanine methylations, 10,000 spontaneous depurinations, 10,000–100,000 oxidative lesions, 10,000 SSBs, and 10–50 DSBs per day [2]. Among these, DSBs represent the most critical and potentially lethal form of DNA damage, as they can lead to mutations, chromosomal aberrations, and cellular dysfunction [3, 4, 5]. Assessing the severity of DSBs relies on two primary parameters: the number of breaks and their physical distribution across the genome. Detection of the physical distribution of DSBs, often referred to as mapping DSBs, is crucial for identifying DSB hotspots (breakome) and understanding the DNA damage and repair processes [6]. The importance of detection for the number of DSBs (N_DSBs_) is also well established with a direct link to the study of DNA damage and repair [7], cell toxicology [8], aging [9], infertility [5], especially to evaluate the impacts of environmental stress, such as occupational exposure, air pollutants, and chemical contaminants, on human health and the environment [10, 11, 12].

Despite the significance of DSBs in environmental health and toxicology, existing detection methods remain either indirect, qualitative, or labor‐intensive, including pulsed‐field gel electrophoresis (PFGE) [13], single cell gel electrophoresis (SCGE) [14], γ‐H2AX assay [15], and SensiTive Recognition of Individual DNA Ends (STRIDE) [16]. Among these, SCGE and γ‐H2AX assay are the most widely used methods. Recently, several methods have been developed for genome‐wide qualitative detection of DSBs, such as i‐BLESS [17], qDSB‐Seq [18] and Induce‐seq [19]. However, these techniques employ next‐generation sequencing, which is expensive, laborious, requires extensive sample preparation, and may introduce biases due to the need for DNA library construction [20].

In response to these challenges, we present a novel, rapid, and cost‐effective approach, named the standard curve method, for the quantitative analysis of whole‐genome N_DSBs_ in humans and four model organisms (mice, *Arabidopsis thaliana* , *Saccharomyces cerevisiae* , and *Escherichia coli* ). This method leverages restriction blunt‐end endonucleases to generate standards with known N_DSB_ levels and uses ligation‐mediated real‐time quantitative PCR (LM‐qPCR) to determine the corresponding Ct values. The resulting standard curves, constructed by plotting log‐transformed N_DSBs_ against Ct values, enable accurate quantification of N_DSBs_ across diverse biological samples. We applied this method to assess N_DSBs_ induced by ionizing radiation (x‐ray) and chemical stress (H_2_O_2_) in human cells and four model organisms, comparing the results with classical DSB detection methods neutral SCGE and γ‐H2AX assay. The proposed method offers a rapid, simple, high‐throughput tool for environmental monitoring, genotoxicity assessment, and DNA repair studies, with the potential to become a standardized kit for large‐scale environmental and public health research.

## 2 Materials and Methods

### 2.1 Theoretical N_DSBs_ of Model Organisms Induced by REases

Whole‐genome DNA sequences of model organisms, including human, mice, *Arabidopsis thaliana* , *Saccharomyces cerevisiae* , and *Escherichia coli* , were obtained from NCBI in FASTA format, excluding mitochondrial DNA and unmapped regions. A specialized software developed in Java, utilizing the string‐matching algorithm “Sunday,” was employed to calculate the frequency of restriction endonuclease (REase) recognition sites within the genome. By inputting the genomic sequence and the REase recognition sequences into the software, we obtained the number of recognition sites and the number of DNA fragments generated by enzymatic cleavage. Assume that REases had *n* recognition sites on one genomic DNA, the theoretical N_DSBs_ of standards were calculated using the following formula:

(1) $$N_{\text{DSBs}}\;\text{of standards}=\text{copy number of genomic}\;\mathrm{DNA}\times n$$

where the copy number of genomic DNA is calculated as:

(2) $$\text{Copy number of genomic}\;\mathrm{DNA}=\frac{\text{mass of genomic}\;\mathrm{DNA}\;\left(\mathrm{\mu g}\right)\times 6.023\times 10^{23}{\mathrm{mol}}^{‐1}}{\text{number of base pairs}\times 10^{6}\times 650}$$

Therefore, theoretical N_DSBs_ for each organism were calculated as follows:

(3) $$N_{\text{DSBs}}\;\text{of human standards}=1.477\times n$$

(4) $$N_{\text{DSBs}}\;\text{of}\;C57\;\mathrm{BL}/\text{6J mice standards}=1.773\times n$$

(5) $$\begin{array}{c} N_{\text{DSBs}}\;\text{of Columbia wild}-\text{type}\;\text{Arabidopsis thaliana}\;\text{standards}\\ =38.88\times n \end{array}$$

(6) $$N_{\text{DSBs}}\;\text{of}\;\text{Saccharomyces cerevisiae}\;S288C\;\text{standards}=761.7\times n$$

(7) $$N_{\text{DSBs}}\;\text{of}\;\text{Escherichia coli}\;\text{ATCC}25922\;\text{standards}=1782\times n$$

### 2.2 Isolation of Genomic DNA From Model Organisms

Human peripheral blood mononuclear cells (PBMC) were provided by healthy volunteers aged 22 to 27 years old from the Third Xiangya Hospital, Central South University, and the genomic DNA was extracted within 24 h. C57 BL/6 J female mice aged 9 weeks were purchased from the Department of Laboratory Animals, Central South University. The whole blood samples were collected from mice eyes, anticoagulated with EDTA‐K_2_, and genomic DNA was extracted within 24 h. The seeds of wild‐type *Arabidopsis thaliana* were provided by the College of Bioscience and Biotechnology of Hunan Agricultural University, and DNA was extracted from tender leaves. *Saccharomyces cerevisiae* S288C was bought from Hangzhou Hongsay Biotechnology Co. Ltd., and genomic DNA was extracted during the logarithmic growth phase. *Escherichia coli* 25,922 was provided by the Central Laboratory of the Department of Laboratory Medicine, Xiangya School of Medicine, Central South University, and DNA was extracted during the logarithmic growth phase. The genomic DNA extraction procedures were performed strictly according to the manufacturer's instructions (Omega Bio‐Tek, USA). The concentration and purity of the extracted genomic DNA were carried out through the NanoDrop One (Thermo Fisher Scientific, USA), and the quality was evaluated by 2% agarose gel electrophoresis. Then the extracted DNA concentration was adjusted to 100 ng/μL and stored at −20°C for further use. Among them, only %Tail DNA < 5% detected by the neutral comet assay of the samples was used for standards preparation.

### 2.3 Preparation of Standards

Genomic DNA extracted from five model organisms (human, mouse, *Arabidopsis*, *Saccharomyces cerevisiae* and *Escherichia coli* ) were digested by seven blunt‐end restriction endonucleases (AluI, BsuRI, DraI, SspI, StuI, EcoRV, EheI) (Thermo Scientific), respectively, to obtain standards with known N_DSB_ levels. In the restriction endonuclease reaction system, according to the instructions, 100 ng of genomic DNA extracted were fully digested by 0.5 μL blunt‐end restriction endonucleases, and 2% agarose gel electrophoresis were performed on the enzyme digestion products to ensure the enzyme digestion reaction completely.

### 2.4 The Establishment of Standard Curves

Before the LM‐qPCR, 50 μmol/L 24 bp 5′‐AGCACTCTCGAGCCTCTCACCGCA‐3′ and 12 bp 5′‐TGCGGTGAGAGG‐3′ unphosphorylated oligonucleotides (Sangon Biotech Co. Ltd.) were mixed and heated in a water bath at 95°C for 3 min and cooled to 25°C within 45 min, followed by a metal bath at 25°C for 10 min to synthesize the double‐strand linkers. Then 2 μL standards, 0.2 nmol double‐strand linkers, 2 U T4 DNA ligase (Thermo Scientific), 2 μL PEG 4000, and 2 μL 10 × Buffer were mixed and added with DEPC water to a final volume of 20 μL, followed by 1 h incubation at 22°C and terminated at 65°C for 10 min. The ligation products were diluted 100 times and stored at −20°C for later use. Finally, the qPCR was performed to detect the Ct values of standards. The optimal reaction system consisted of 2 μL diluted ligation products, 0.03 nmol primers (24 bp ligonucleotides, 5′‐AGCACTCTCGAGCCTCTCACCGCA‐3′), 12 μL 2 × KOD SYBR qPCR Mix (TOYOBO), and added with DEPC water to 20 μL.

The ligation products without T4 DNA ligase and amplification template were used as negative controls. The quality of the LM‐qPCR amplification products were detected by 2% agarose gel electrophoresis. Under the optimal reaction conditions, LM‐qPCR had a good amplification efficiency when DNA fragment size < 2000 bp, so the theoretical N_DSBs_ of standards were calculated using the number of < 2000 bp fragments. According to the measured Ct values and the calculated theoretical N_DSBs_ of standards, the Ct‐lgN_DSBs_ standard curves and the fitting equations could be constructed. Hence, by detecting the Ct value of the unknown sample, N_DSBs_ could be quickly be determined.

### 2.5 Repeatability, Sensitivity, and Specificity Tests of Standard Curve Method

To assess repeatability, the Ct values of standards were measured through parallel experiments, calculating inter‐assay and intra‐assay coefficients of variation (CV). The human genomic DNA was digested by EheI for 5 min, 30 min, and 1 h, respectively, to obtain the samples with low, medium, and high N_DSBs_, and these samples were detected by the standard curve method and the inter‐assay and intra‐assay CV values were calculated to verify the repeatability of the experiment.

For sensitivity and specificity, human genomic DNA was digested with EcoRV and the resulting products serially diluted 10–10^6^ times after ligation reaction. After dilution, the samples were detected, and Ct‐lgN_DSBs_ curves were established to test the sensitivity and specificity of the standard curve method. At the same time, we repeated the detection of the blank group to get the lowest detection limit of this method.

### 2.6 Quantitative Analysis of N_DSBs_ Induced by X‐Ray and H_2_O_2_

Before testing, sticky‐ends of the DNA fragments must be flattened, since only blunted‐ends DNA fragments can be connected by LM‐qPCR. Klenow fragment (Thermo Scientific) and T4 DNA polymerase (Thermo Scientific) were used to flatten the sticky‐ends, respectively. The human peripheral blood mononuclear cells (PBMC) and the whole blood of mice were irradiated with 0, 2, 4, 8 and 12Gy x‐ray or treated with 0, 20, 40, 60 and 100 μmol/L H_2_O_2_ for 1 h. *Arabidopsis thaliana* leaf lapping liquid, *Saccharomycetes cerevisiae* and *Escherichia coli* were treated with 0, 2, 4, 6 and 10 mmol/L H_2_O_2_ for 1 h. After treatment, genomic DNA were extracted and the sticky‐ends of the DNA fragments were flattened. Then LM‐qPCR was used to detect Ct values of the above treated samples.

### 2.7 Neutral SCGE and γ‐H2AX Assay

Neutral SCGE was performed to detect the degree of DSBs in human PBMCs and C57 BL/6 J mice whole blood cells after the above treatment, using the protocol described by Dunkenberger [14]. Flow cytometry for γ‐H2AX was used to detect the degree of DSBs in the treated human PBMC. PBMC were isolated from 2 mL human peripheral blood and treated as above treatment. The cells were fixed with 4% paraformaldehyde for 20 min and permeabilized with 0.5% TritonX‐100 at room temperature for 15 min. Then the cells were incubated in FITC‐labeled mouse anti‐human γ‐H2AX antibody (eBioscience, 1/100 dilution) or PBS (as negative control) for 1 h in dark. In the end, the expressions of γ‐H2AX were detected by flow cytometry. The positive cell rate was obtained, and the average geometric fluorescence intensity was counted using FlowJo 7.6 software.

### 2.8 Statistical Analysis

Results of quantification are shown as mean ± SD and each experiment was performed three times. Statistical analysis was performed by SPSS 24.0 and GraphPad 7.0. One‐way analysis of variance was used for comparison of quantitative data among multiple groups, and LSD‐t test was used for pair comparison between groups. Pearson correlation test was used for correlation analysis and the correlation coefficient was expressed as *r*. *p* < 0.05 were considered to be statistically difference.

## 3 Results

### 3.1 Theoretical N_DSBs_ of Model Organisms Induced by REases

The software developed for this study, named Specific DNA Sequence Finder (Figure S1), is designed to identify the recognition sequences of seven blunt‐end restriction enzymes (REases), as shown in S1. The software, which utilizes the “Sunday” string‐matching algorithm, could calculate the number of recognition sites and the number of DNA fragments generated after enzyme digestion in genomic DNA within 30s. By inputting the genomic sequence and REase recognition sites into the software, the theoretical N_DSBs_ of the five biological standards were determined (Table 1). Notably, EheI, which is sensitive to CpG methylation in mammals, was excluded from the subsequent construction of standard curves for human and mice due to potential inaccuracies in those species.

**TABLE 1:** The theoretical numbers of DSBs of standards cut with blunt‐terminal restriction endonucleases.

Biological type	Restriction endonuclease						
	AluI	BsuRI	DraI	SspI	StuI	EcoRV	EheI
Human	37,817,821	24,554,816	8,910,629	5,654,455	1,500,820	396,760	208617*
C57mice	43,178,529	23,244,558	7,369,625	4,028,286	1,112,607	479,758	63204*
*Arabidopsis thaliana*	37,562,901	8,167,522	9,344,652	7,827,944	148,599	1,038,329	22,395
*Saccharomyces cerevisiae*	30,195,311	13,964,246	5,479,670	7,428,098	239,174	1,649,081	115,778
*Escherichia coli*	26,227,476	24,682,482	1,958,418	2,740,716	306,504	2,544,696	90,882

* These data were excluded from the subsequent construction of the standard curves.

### 3.2 Preparation and Validation of Standards

**FIGURE 1:**
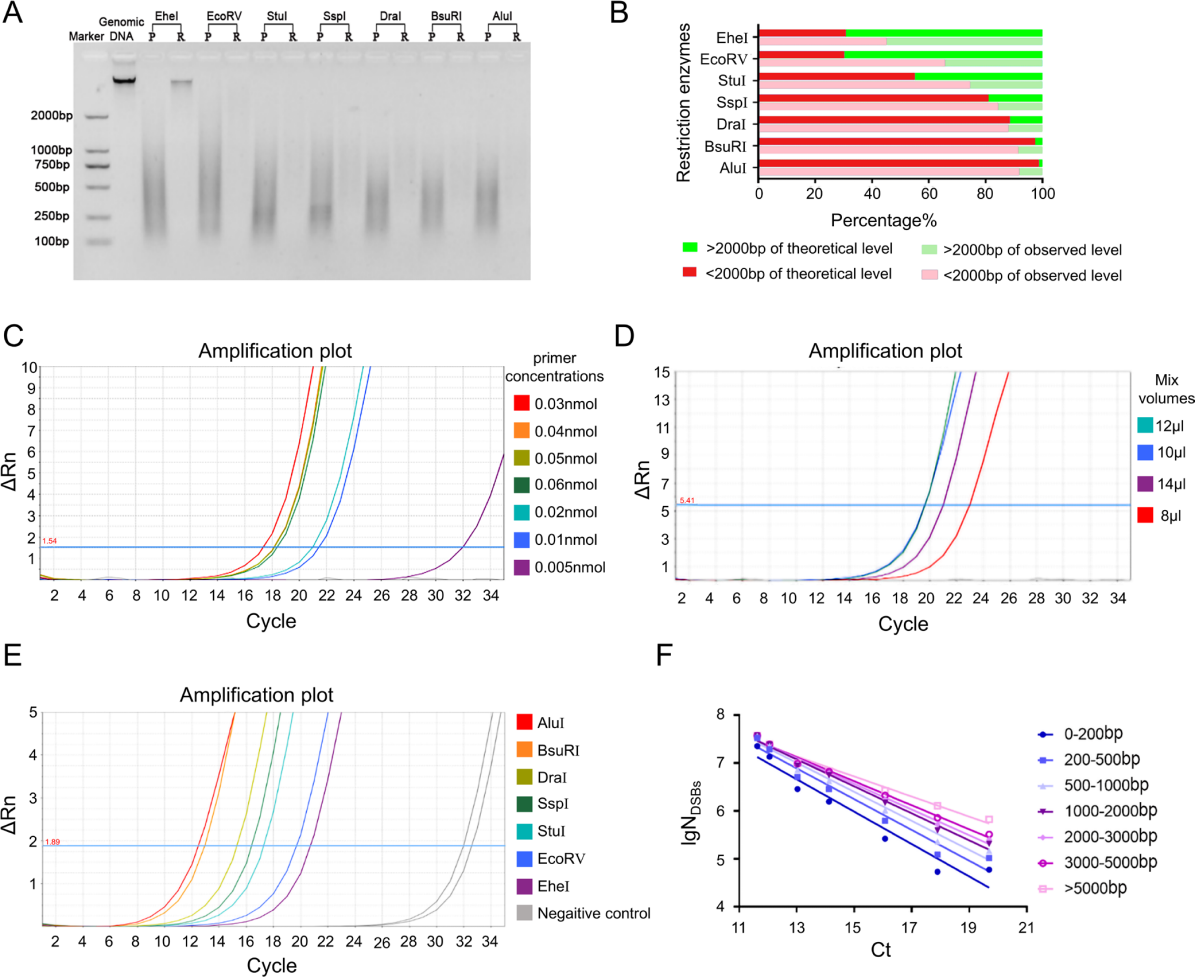
The condition optimization and establishment of the standard curve method in human standards. (A) Agarose gel electrophoresis result of human genomic DNA extraction products, LM‐qPCR amplification products, and enzyme digestion products. P: The LM‐qPCR amplification products; R: The enzyme digestion products. (B) Comparison of theoretical and experimental DNA fragments after restriction enzyme digestion of human genomic DNA. (C) LM‐qPCR amplification curves of the same sample with different primer concentrations. From left to right, the final primer concentrations were 0.03, 0.04, 0.05, 0.06, 0.02, 0.01, and 0.005 nmol, respectively. (D) LM‐qPCR amplification curves of the same sample with different volumes of Mix. From left to right, the volumes of Mix were 12, 10, 14, and 8 μL, respectively. (E) The LM‐qPCR amplification curve of human standards. (F) The Ct‐lgN_DSBs_ (different size DNA fragments) standard curves of human standards.

The genomic DNA of the five model organisms was digested by the seven REases to generate standards with known N_DSBs_. The optimal digestion times of AluI, DraI, SspI, StuI, BsuRI, EcoRV and EheI were 30 min, 5 min, 30 min, 5 min, 2 h, 4 h and 1 h, respectively (the data didn't shown). Then 2% agarose gel electrophoresis were performed to assess the quality of DNA products. The results were shown in Figure 1A and Figure S2. Among them, the bands of genomic DNA extraction products were linear, explained that the quality of extracted genomic DNA were perfect, and the bands of enzyme digestion products were diffuse, due to the random identified sites of REases caused different size DNA fragments. The percentages of the gray value of bands which were < 2000 bp in the corresponding electrophoresis zones gray value were calculated, and Pearson correlation analysis was conducted between the results and the theoretical percentages of < 2000 bp recognition sequence number by the software (Figure 1B and Figure S2). Correlation coefficient r were all greater than 0.95, which proved the reliability of standards, so the theoretical N_DSBs_ of standards could reflect the real N_DSBs_ level.

### 3.3 Establishment of Standard Curves

**FIGURE 2:**
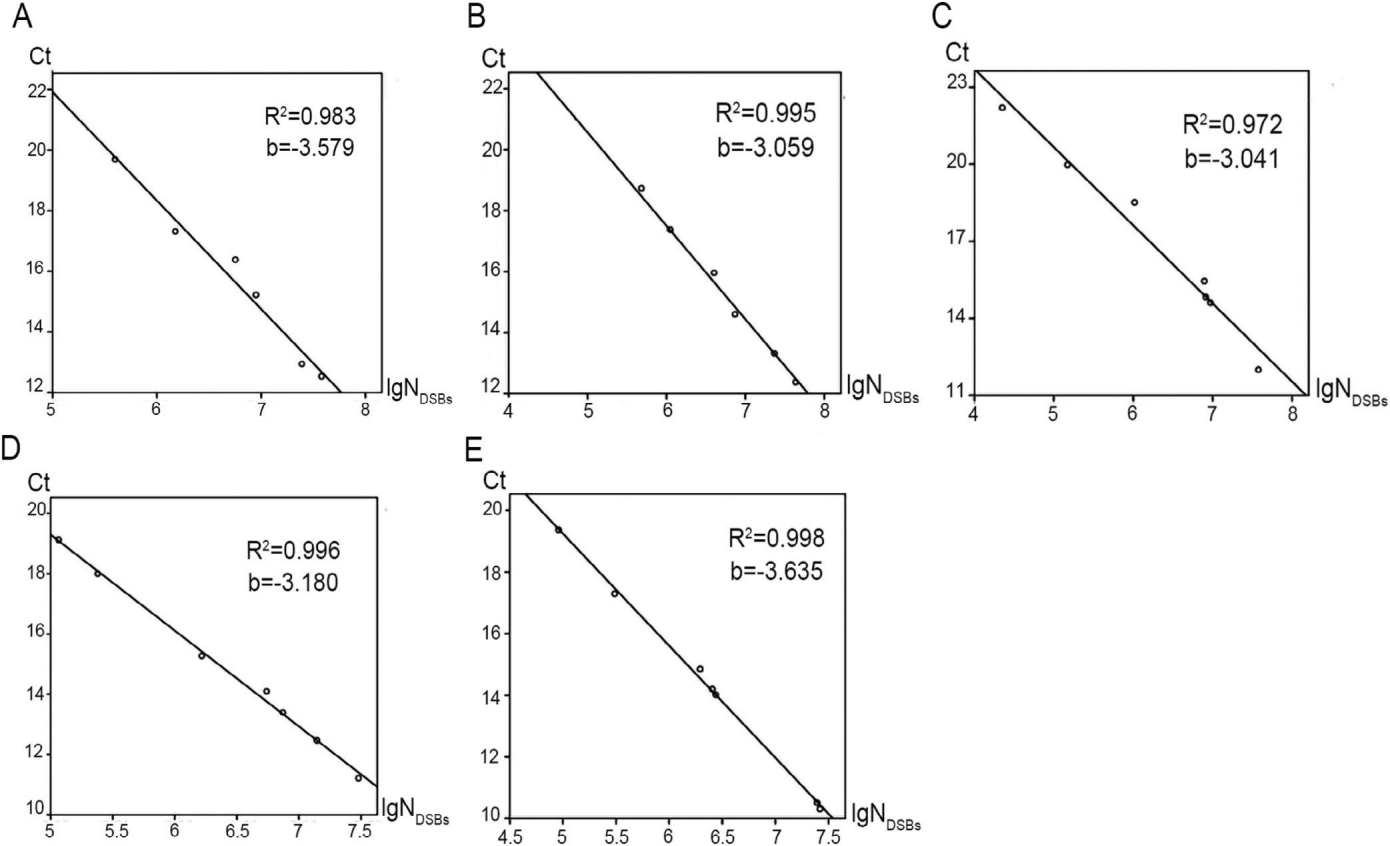
The Ct‐lgN_DSBs_ standard curves of human and model organisms standards. (A) standard curve of human. (B) standard curve of mice. (C) standard curve of *Arabidopsis thaliana* . (D) standard curve of *Saccharomyces cerevisiae* . (E) standard curve of *Escherichia coli* .

The LM‐qPCR reaction system contain 0.03 nmol primer and 12 μL 2 × KOD SYBR qPCR Mix (Figure 1C,D), with the optimal reaction conditions which were shown in Table S2. Under the optimal reaction conditions, the results of standards Ct values were shown in Table 2. The theoretical N_DSBs_ for different‐sized DNA fragments were calculated, and the fitting curves were established according to the Ct value detected by LM‐qPCR and the N_DSBs_ (Figure 1E,F and Figure S3). The results demonstrated a strong linear relationship between the N_DSBs_ of DNA fragments < 2000 bp and the Ct values for all five organisms. Therefore, N_DSBs_ of DNA fragment < 2000 bp and their Ct values were used to establish the standard curves. Due to the CpG methylation sensitivity, EheI was excluded from the standard curve construction for human and mouse samples (Figure 2). As the Ct values of the standards showed a linear trend, the fitting equations and parameters could be obtained by linear curve fitting (Table 3). The R^2^ of the fitting equations were all > 0.95, indicated the existence of a strong correlation between Ct values and the lgN_DSBs_. The slope (b) of curves was between 3.0 to 3.65, indicating amplification effciency was 87.9% to 115.4%.

**TABLE 2:** The Ct values of five biological standards.

Biological type	Restriction endonuclease						
	AluI	BsuRI	DraI	SspI	StuI	EcoRV	EheI
Human	12.302	12.726	15.026	16.155	17.109	19.460	20.534*
C57mice	12.382	13.324	14.604	15.965	17.376	18.732	20.928*
*Arabidopsis thaliana*	12.006	14.835	14.617	15.456	19.980	18.519	22.202
*Saccharomyces cerevisiae*	11.213	12.469	14.098	13.396	17.998	15.271	19.125
*Escherichia coli*	10.313	10.515	14.851	14.018	17.293	14.199	19.368

* These data were excluded from the subsequent construction of the standard curves.

**TABLE 3:** The fitting equations and parameters of human and model organisms.

Biological type	Standard equations	*R* ^2^	|*b*|	Average intra‐assay CV	Average inter‐assay CV
Human	Ct = 39.846 − 3.586 lgN_DSBs_	0.983	3.586	1.092%	6.119%
C57mice	Ct = 35.851 − 3.059 lgN_DSBs_	0.995	3.059	3.300%	2.467%
*Arabidopsis thaliana*	Ct = 37.414 − 3.635 lgN_DSBs_	0.998	3.635	2.446%	7.500%
*Saccharomyces cerevisiae*	Ct = 35.192 − 3.180 lgN_DSBs_	0.996	3.180	1.477%	2.921%
*Escherichia coli*	Ct = 35.872 − 3.041 lgN_DSBs_	0.972	3.041	2.154%	5.263%

### 3.4 Repeatability, Sensitivity, and Specificity of the Standard Curve Method

**FIGURE 3:**
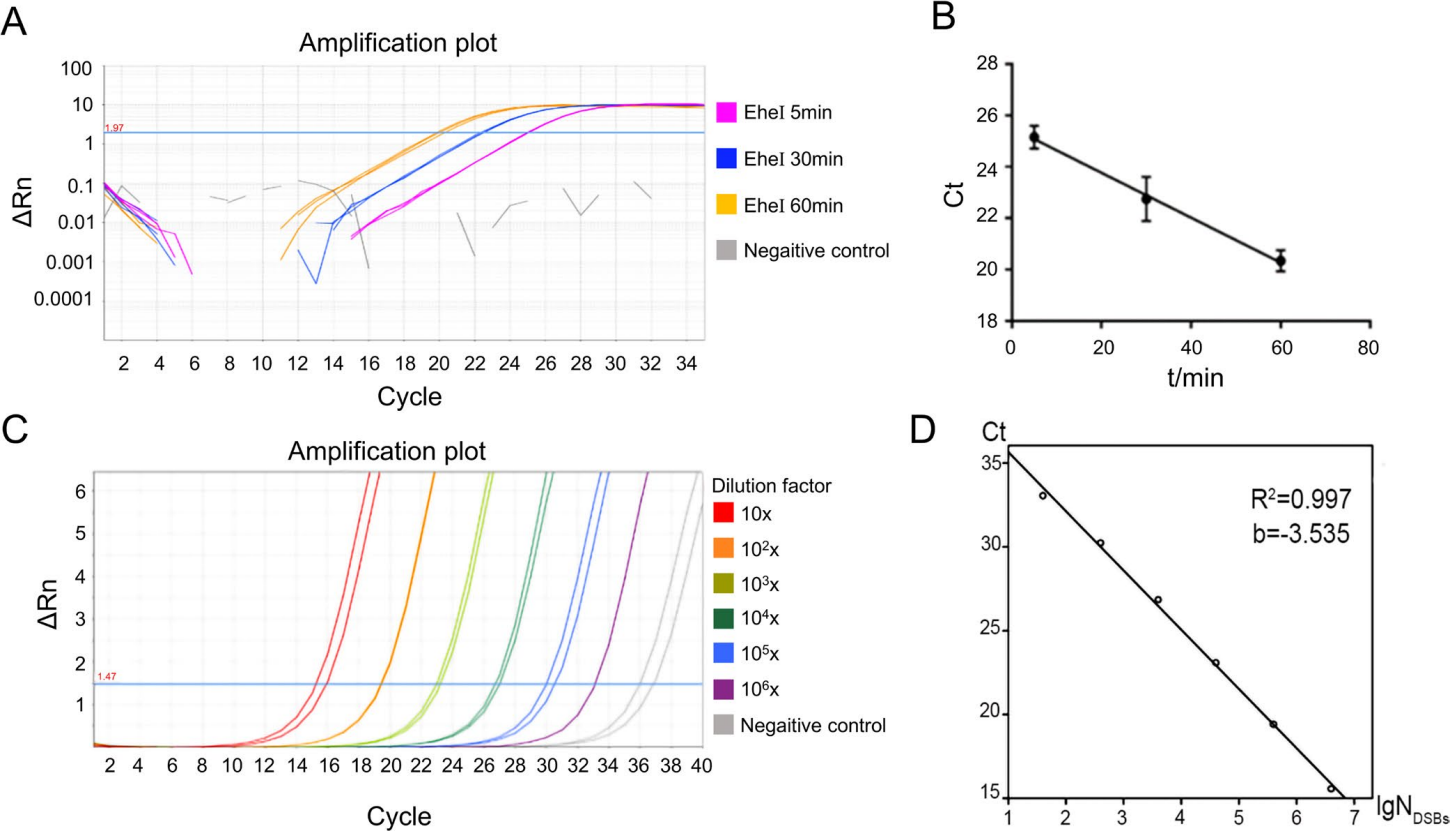
The repeatability, sensitivity, and specificity tests of the standard curve method. (A) LM‐qPCR amplification curves of human genomic DNA treated with EheI for different times: 5, 30, and 60 min, from left to right. (B) Fitting curve of EheI digestion time and Ct value. (C) The LM‐qPCR amplification curve after 10–10^6^ times dilution of human standards treated with EcoRV. (D) The fitting curve after 10–10^6^ times dilution of human standards.

Through the repeatability measurements of the standards, the average values of intra‐assay CV were all less than 3%, and the average values of inter‐assay CV were all less than 7.5% (Table 3). And the intra‐assay CV of the samples with low, medium, and high N_DSBs_ were 0.05%, 0.43% and 1.1%; the inter‐assay CV were 1.78%, 3.78% and 2.03%, respectively. The average intra‐assay CV was 0.53% and the average inter‐assay CV was 2.53%. All these results showed that the standard curve method had good reproducibility for samples with different N_DSBs_ levels (Figure 3A,B). After 10–10^6^ times dilution of standards, the ligation products still had good amplification curves, reminded a good sensitivity (Figure 3C,D and Figure S4). In addition, the slopes of the fitting curves were between 3.0 to 3.8, and the Ct value of the control group without template or T4 DNA ligase were all greater than 33 or none. Therefore, the standard curve method could detect the N_DSBs_ damage in the range of 10 to 10^8^, and showed a good sensitivity and specificity. Besides, by repeatedly detecting the control group, we obtained that the minimum detection limit of this method was 17.62.

### 3.5 Detection of DSBs Induced by X‐Ray and H_2_O_2_

**FIGURE 4:**
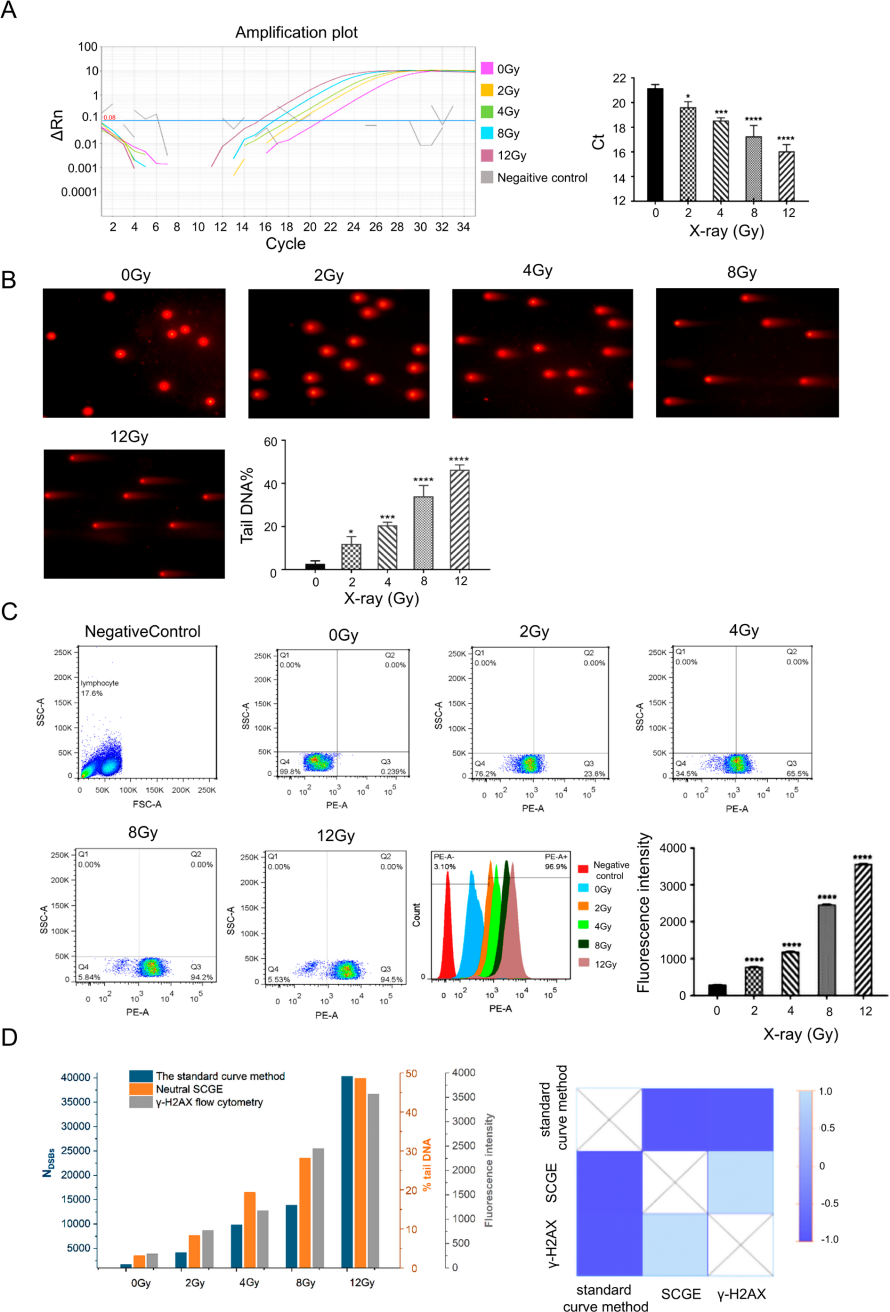
Detection of DSBs in human PBMCs induced by x‐ray using the standard curve method, neutral SCGE, and γ‐H2AX flow cytometry. (A) The standard curve method results of DSBs induced by x‐ray. (B) Neutral SCGE results of DSBs induced by x‐ray. (C) γ‐H2AX flow cytometry results of DSBs induced by x‐ray. (D) Analysis of results of DSBs induced by x‐ray from three methods and the heat map of the correlation analysis. *p < 0.05, **p < 0.01, ***p < 0.001, ****p < 0.0001; for each group, *n* = 3.

**FIGURE 5:**
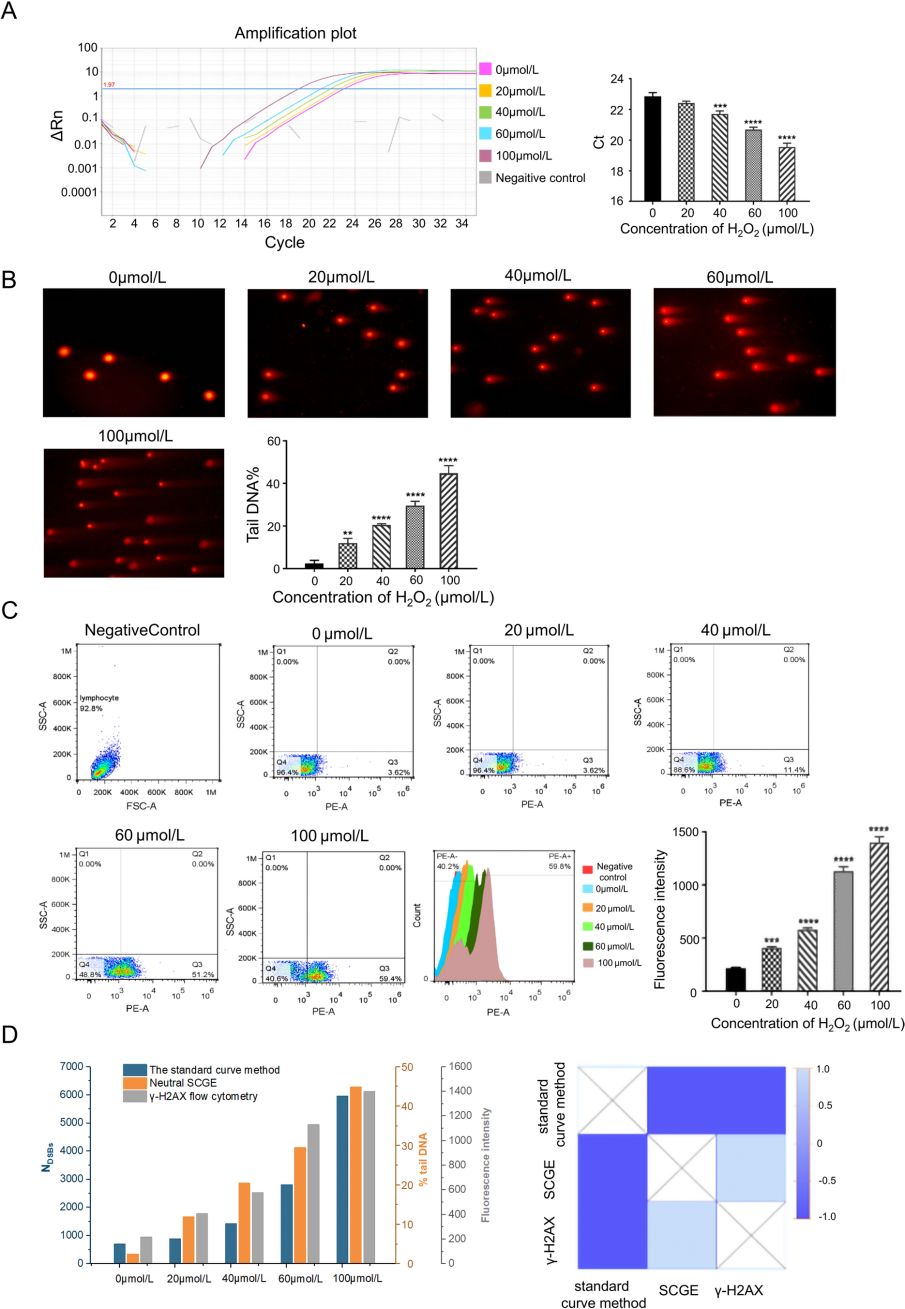
Detection of DSBs in human PBMCs induced by H_2_O_2_ using the standard curve method, neutral SCGE, and γ‐H2AX flow cytometry. (A) The standard curve method results of DSBs induced by H_2_O_2_. (B) Neutral SCGE results of DSBs induced by H_2_O_2_. (C) γ‐H2AX flow cytometry results of DSBs induced by H_2_O_2_. (D) Analysis of results of DSBs induced by H_2_O_2_ from three methods and the heat map of the correlation analysis. *p < 0.05, **p < 0.01, ***p < 0.001, ****p < 0.0001; for each group, *n* = 3.

To assess the applicability of the standard curve method, we quantified N_DSBs_ induced by x‐ray irradiation and H₂O₂ treatment. However, DSBs induced by x‐ray or H₂O₂ may produce blunt‐ or sticky‐ends DNA fragments, while the linkers used in LM‐PCR can only be connected with blunt‐ends. Therefore, Klenow fragment and T4 DNA polymerase were used to flatten the sticky‐ends of DSBs separately and evaluated by LM‐qPCR (Figure S5). Among them, the sample flattened by Klenow fragment showed the lower Ct value means the effect of Klenow fragment was best. So, Klenow fragment was used to in the subsequent detection. We used the standard curve method to detect the N_DSBs_ caused by different doses of x‐rays (Figure 4A and Figure S6A). With the increase dose of x‐ray, the Ct value decreased in a dose‐dependent manner, indicated that the N_DSBs_ increases, and the differences had statistically significant (human: *F* = 42.18, mice: *F* = 42.66, *p* < 0.0001). The standard curve method was also employed to detect N_DSBs_ caused by different concentrations of H_2_O_2_ (Figure 5A and Figure S7). With the increase concentration of H_2_O_2_, the Ct value decreased means the N_DSBs_ increased, and the differences were statistically significant (human: *F* = 138.8, mouse: *F* = 37.36, *Arabidopsis thaliana* : *F* = 38.79, *Saccharomyces cerevisiae* : *F* = 19.83, *Escherichia coli* : *F* = 17.14, *p* < 0.001). Simultaneously, neutral SCGE was performed to detect the DSBs of above x‐ray and H_2_O_2_ treated samples (Figures 4B and 5B; Figures S6B and S8A). With the increase dose of x‐ray and concentrations of H_2_O_2_, the “comet” Tail increased, and the %Tail DNA increased in a dose‐dependent manner, which indicated that the degree of DSBs increased (x‐ray: *F* = 99.36 in human, *F* = 416 in mice; H_2_O_2_: *F* = 168.2 in human, *F* = 228.7 in mice, *p* < 0.0001). The degree of DSBs induced by x‐rays and H_2_O_2_ in human samples were also tested by γ‐H2AX flow cytometry (Figures 4C and 5C). With the increase of x‐ray dose and H_2_O_2_ concentration, the positive rate of γ‐H2AX (Q2 + Q3) detected increased, and the peak value of γ‐H2AX fluorescence intensity shifted to the right, which indicated that the degree of DSBs increased (x‐ray: *F* = 57,296, H_2_O_2_: *F* = 735.5, *p* < 0.0001).

Simultaneously, neutral SCGE was performed to detect the DSBs of above x‐ray and H_2_O_2_ treated samples (Figures 4B and 5B; Figures S6B and S8A). With the increase dose of x‐ray and concentrations of H_2_O_2_, the “comet” Tail increased, and the %Tail DNA increased in a dose‐dependent manner, which indicated that the degree of DSBs increased (x‐ray: *F* = 99.36 in human, *F* = 416 in mice; H_2_O_2_: *F* = 168.2 in human, *F* = 228.7 in mice, *p* < 0.0001). The degree of DSBs induced by x‐rays and H_2_O_2_ in human samples were also tested by γ‐H2AX flow cytometry (Figures 4C and 5C). With the increase of x‐ray dose and H_2_O_2_ concentration, the positive rate of γ‐H2AX (Q2 + Q3) detected increased, and the peak value of γ‐H2AX fluorescence intensity shifted to the right, which indicated that the degree of DSBs increased (x‐ray: *F* = 57,296, H_2_O_2_: *F* = 735.5, *p* < 0.0001).

### 3.6 Correlation Between the Standard Curve Method, Neutral SCGE, and γ‐H2AX Flow Cytometry

The correlation between the three methods (standard curve method, neutral SCGE, and γ‐H2AX flow cytometry) was evaluated using Pearson correlation analysis. The absolute values of the correlation coefficients (r) were all greater than 0.9, indicating a strong positive correlation between the N_DSBs_ detected by the standard curve method and the results from both SCGE and γ‐H2AX flow cytometry (Figures 4D and 5D; Figures S6C and S8B). These results demonstrate the reliability and accuracy of the standard curve method for quantifying DSBs, with potential applications in environmental exposure monitoring and genotoxicity studies.

## 4 Discussion

Quantifying the amount of DNA double‐strand breaks is crucial for assessing the exposure levels of organisms to the genotoxic effects of environmental contaminants. In this study, we developed a simple, rapid and sensitive method, termed the standard curve method, for the quantitative analysis DSBs number in genome DNA. This method is applicable to a wide range of organisms and sample types, making it highly relevant for environmental monitoring and human health risk assessments. We validated this method in human, mouse, *Arabidopsis*, *Saccharomyces cerevisiae* and *Escherichia coli* , and compared it with two classic DSBs detection methods. Our method offers several unique features and advantages over existing assays. First, it enables quantitative analysis of DSBs using a standard curve framework. Second, the method exhibits high sensitivity and a wide detection range, which is critical when assessing DSBs induced by various endogenous and exogenous agents, including pollutants and ionizing radiation. Third, it is simple, cost‐effective, and rapid, requiring only basic thermal cycling equipment. Lastly, this method is applicable not only to fully sequenced genomes but also to sequenced DNA fragments, enhancing its versatility.

A key innovation of this method is the preparation of standards. Multiple blunt‐end REases were used to cleave genomic DNA, thereby generating standards with known DSB numbers. With the development of high throughput sequencing technologies, more than 60,000 species of genome sequence information can be obtained from the NCBI website [21], which makes it possible to quantify the numbers of specific DNA sequences in genomic DNA. So, we designed a custom software which could efficiently scans for specific REases recognition sequences and calculates the theoretical number of DSBs. LM‐PCR use ligation reaction to connect asymmetric common double‐stranded DNA adapter at both ends of DNA fragments, and amplifies with the adapter primers [22]. By combining ligation‐mediated PCR (LM‐PCR) with real‐time quantitative PCR (qPCR), we established LM‐qPCR that monitors fluorescence accumulation during amplification. In the exponential phase, a higher number of starting templates corresponds to lower Ct values, which served as the quantitative basis of our method.

An important technical consideration is that only blunt‐end REases were employed to generate standards, as the common linkers in LM‐PCR require blunt ends. In unknown samples, DSBs induced by endogenous or exogenous agents predominantly produce blunt‐ and sticky‐end DNA fragments. To convert sticky‐end fragments into blunt ends, Klenow fragment or T4 DNA polymerase is commonly used [23, 24]. So, in this study, we also assessed the effectiveness of flattening sticky ends from DSBs in unknown samples and determined that treatment with the Klenow fragment was the most effective. However, it is important to note that although Klenow efficiently processes classical two‐ended DSBs, it is not suitable for more complex break structures such as single‐ended DSBs (seDSBs) generated by replication stress or specific nucleases' cleavage. These complex structures may be underestimated by our method, but seDSBs are more relevant to specialized replication stress models than to general environmental or toxicological exposures, which is the main application of our method. Hence, this limitation does not compromise the primary applications of our method. Future adaptations could incorporate additional enzymatic or cell‐based approaches to specifically process such complex DSB structures and improve their quantification.

Chromatin structure and accessibility in eukaryotic cells may also potentially affect the efficiency of linker ligation in LM‐qPCR and thus affect DSB detection [25]. Tightly packed heterochromatin can hinder linker accessibility and reduce detection efficiency, while euchromatic regions are more readily detected. Although our method quantifies total DSB load rather than their distribution, such differences may introduce minor biases in complex genomes. Future work may mitigate this limitation by integrating chromatin accessibility maps or by optimizing linker design.

In addition, some blunt‐end REases could be methylation sensitive, which could cause discrepancies between the observed and theoretical N_DSBs_. Among the seven blunt‐end REases used in this study, only EheI is CpG methylation sensitive in mammals. Thus, EheI was excluded from the subsequently constructed standard curves of human and mice. However, in Figure 1A,B and Figure S2, our validation experiments showed a strong correlation between theoretical and observed DSB numbers generated by EheI both in human and mice, suggesting that the CpG methylation has a limited impact on the overall quantification under our conditions. As a result, we still used EheI to verify the repeatability of the standard curve method. Nevertheless, further investigation is required to fully elucidate the impact of methylation on this method.

At present, neutral SCGE and γ‐H2AX assays are the most widely used methods for detecting DSBs. Neutral SCGE, also known as the comet assay, depends on the relaxation of nucleoid DNA in agarose forming a comet‐like image [26]. The relative amount of DNA in the comet tail indicates the degree of DSBs. γ‐H2AX is widely recognized as a sensitive biomarker of cellular response and serves as an indirect indicator of DSBs. The detection of γ‐H2AX relies on the rapid phosphorylation of serine histone H2AX to form γ‐H2AX after DSBs [27]. The comet assay suffers from significant inter‐ and inter‐laboratory variability [28, 29], and γ‐H2AX assays, despite their sensitivity, are limited by low resolution and potential false positives [30, 31]. In contrast, our method demonstrated excellent repeatability with intra‐assay and inter‐assay coefficients of variation of 0.527% and 2.528%, respectively. It also exhibited high sensitivity (with a detection limit of 17.62 DSBs) and a broad detection range (from 10 to 10^8^ DSBs), making it a robust tool for assessing genotoxic effects across diverse sample types. Furthermore, this method requires only simple operations and equipment, with a detection time of less than 4 h after the preparation of the standards. The applicability of the standard curve method was also validated by quantifying DSBs induced by x‐ray irradiation and H_2_O_2_ treatment in human cells, mice, *Arabidopsis thaliana* , *Saccharomyces cerevisiae* , and *Escherichia coli* . The observed dose‐dependent increase in DSBs correlated strongly with results obtained from both neutral SCGE and γ‐H2AX assays, underscoring the reliability of our approach for environmental and toxicological studies. In addition, neutral SCGE was primarily used for DSB analysis in eukaryotic cells [32, 33, 34], while γ‐H2AX assays were mainly employed in human cells exposed to ionizing radiation and genotoxic agents. In our study, the standard curve method was proved to be applicable to all DNA samples with known sequences, including both prokaryotes and eukaryotes, demonstrating its broader applicability across diverse research contexts.

It is well‐known that it is impossible to detect the number of DSBs accurately because of DNA frangibility. So, the standard curve method to quantify the N_DSBs_ actually is not quantifying total DSB numbers, but rather a relative fraction of fragments. Meanwhile, restriction sites are not uniformly distributed across genomes, which may lead to slight deviations between theoretical and actual DSB numbers. However, in Figure 1A,B and Figure S2, we observed a strong positive correlation between the theoretical N_DSBs_ and observed N_DSBs_ (*r* > 0.95) across five organisms, suggesting that our approach provides a reliable proxy for total DSBs load, and the impact of uneven site distribution is limited under our experimental conditions, likely because the use of multiple enzymes with diverse recognition sequences partially mitigates this bias. Furthermore, the strong correlation of our quantification results with those from neutral comet assay and γ‐H2AX flow cytometry, which are sequence‐distribution independent, also demonstrates that the potential impact on overall quantification accuracy is minimal.

The efficiency of qPCR is negatively affected by amplicon length, as longer DNA fragments are typically amplified with lower efficiency than shorter targets [35, 36]. In this study, we adopted a two‐step qPCR that reduces template length constraints and improves amplification efficiency. We calculated the corresponding theoretical N_DSBs_ based on the size of different DNA fragments and found that the < 2000 bp theoretical N_DSBs_ showed a robust linear relationship with the Ct value in different organisms (Figures 1F and 2). So, our analysis focused on the number of DNA fragments < 2000 bp obtained from the software for calculating the theoretical N_DSBs_ (Table 1). Fortunately, studies have shown that the physical distribution of DSBs in the genome is not average, but concentrated in specific heritable points, called chromosomal fragile sites (CFSs), which have more physiological and clinical relevance [37, 38]. Therefore, we think that this limitation will not have a great impact on its practical applications. But it should be noted that although our two‐step qPCR approach alleviates this problem, very large DNA fragments (> 2000 bp) may still be underrepresented, leading to a slight bias toward DSB quantification. Additional optimization may be needed for samples enriched in unusually long fragments.

Finally, it is also important to emphasize that the standard curve method is specific to detect DSBs and cannot map them or identify other types of DNA damage such as oxidative base modifications or single‐strand breaks. This defines the practical scope of the method: it is best suited for quantitative assessment of total DSB load rather than mapping or characterizing diverse DNA damage types. However, integration with complementary assays, such as the FPG‐modified comet assay for oxidized purines or sequencing‐based mapping approaches [39], could broaden its utility.

## 5 Conclusion

In summary, the standard curve method proposed in this study represents a significant advancement for quantifying DSBs with high sensitivity, reproducibility, and broad applicability. Its simplicity, low cost and minimal equipment requirements make it an ideal tool for early cancer screening, aging, environmental monitoring, genotoxicity assessments, and public health risk evaluations.

## Author Contributions

L.G.: Formal analysis, investigation, methodology, writing – original draft, writing – review and editing. H.D.: Data curation, formal analysis, methodology, writing – original draft, writing – review and editing. J.L.: Methodology, software, writing – review and editing. C.L.: Methodology, writing – review and editing. Y.H.: Methodology, writing – review and editing. K.X.: Conceptualization, funding acquisition, investigation, project administration, supervision, writing – review and editing.

## Ethics Statement

Human specimens utilized in this study were approved by the Ethics' Committee of The Third Xiangya Hospital of Central South University (approval number: 2023‐R19043) in accordance with the principles of the Declaration of Helsinki. Written informed consent was secured from every participant. And all animal experiments were conducted in accordance with the ARRIVE guidelines, and protocols were approved by the Institutional Animal Care and Use Committee (IACUC) of Central South University (approval number: CSU‐2023‐084).

## Conflicts of Interest

The authors declare no conflicts of interest.

## Supporting information


**Figure S1:** The interface figure of the software “Specific DNA Sequence Finder”. (A) The software interface figure. (B)The identification result of restriction enzyme AluI on human X chromosome DNA by software.
**Figure S2:** The results of agarose gel electrophoresis and gray value analysis of enzyme digestion extraction products in model organisms. (A) mice. (B) *Arabidopsis thaliana* . (C) *Saccharomyces cerevisiae* . (D) *Escherichia coli* . P: the LM‐qPCR amplification products; R: the enzyme digestion products.
**Figure S3:** The LM‐qPCR amplification curves and the Ct‐lgNDSBs (different DNA size fragements) standard curves of model organisms standards. (A) mice. (B) *Arabidopsis thaliana* . (C) *Saccharomyces cerevisiae* . (D) *Escherichia coli* .
**Figure S4:** The LM‐qPCR amplification curves and the fitting curves after 10–10^6^ times dilution of model organisms standards. (A) mice. (B) *Arabidopsis thaliana* . (C) *Saccharomyces cerevisiae* . (D) *Escherichia coli* .
**Figure S5:** LM‐qPCR amplification curves of using Klenow fragment, T4 DNA polymerase or not to flatten the sticky ends.
**Figure S6:** Detection of DSBs in mice whole blood induced by x‐ray using the standard curve method and neutral SCGE. (A) The standard curve method results of DSBs induced by x‐ray. (B) Neutral SCGE results of DSBs induced by x‐ray. (C) Analysis of results of DSBs induced by x‐ray from two methods and the heat map of the correlation analysis. * *p* < 0.05, ** *p* < 0.01, *** *p* < 0.001, **** *p* < 0.0001; for each group, *n* = 3.
**Figure S7:** The standard curve method results of model organisms samples treated by H_2_O_2_. (A) mice. (B) *Arabidopsis thaliana* . (C) *Saccharomyces cerevisiae* . (D) *Escherichia coli* .
**Figure S8:** Detection of DSBs in mice whole blood induced by H_2_O_2_. (A) The neutral SCGE results of mice samples treated by H_2_O_2_. (B) Analysis of results of DSBs induced by H_2_O_2_ from standard curve method and neutral SCGE and the heat map of the correlation analysis.

**Table S1:** The recognized sequence of seven blunt‐terminal restriction endonucleases.
**Table S2:**. The reaction condition of LM‐qPCR.
**Table S3:** The results of human PBMCs treated with x‐ray.
**Table S4:** The results of human PBMCs treated with H_2_O_2_.
**Table S5:** The results of mice sample treated with x‐ray.
**Table S6:** The results of mice sample treated with H_2_O_2_.

## Data Availability

Data will be made available on request.
