# Supplementary material for: Quantitative Analysis of DNA Double‐Strand Breaks in Genomic DNA Using Standard Curve Method
Source: J Clin Lab Anal. 2025 Oct 18;39(23):e70123. doi: 10.1002/jcla.70123 (PMC12699195; doi:10.1002/jcla.70123)
Supplement: Supplementary file 1 — Figure S1: The interface figure of the software “Specific DNA Sequence Finder”. (A) The software interface figure. (B)The identification result of restriction enzyme AluI on human X chromosome DNA by software. Figure S2: The results of agarose gel electrophoresis and gray value analysis of enzyme digestion extraction products in model organisms. (A) mice. (B) Arabidopsis thaliana . (C) Saccharomyces cerevisiae . (D) Escherichia coli . P: the LM‐qPCR amplification products; R: the enzyme digestion products. Figure S3: The LM‐qPCR amplification curves and the Ct‐lgNDSBs (different DNA size fragements) standard curves of model organisms standards. (A) mice. (B) Arabidopsis thaliana . (C) Saccharomyces cerevisiae . (D) Escherichia coli . Figure S4: The LM‐qPCR amplification curves and the fitting curves after 10–106 times dilution of model organisms standards. (A) mice. (B) Arabidopsis thaliana . (C) Saccharomyces cerevisiae . (D) Escherichia coli . Figure S5: LM‐qPCR amplification curves of using Klenow fragment, T4 DNA polymerase or not to flatten the sticky ends. Figure S6: Detection of DSBs in mice whole blood induced by x‐ray using the standard curve method and neutral SCGE. (A) The standard curve method results of DSBs induced by x‐ray. (B) Neutral SCGE results of DSBs induced by x‐ray. (C) Analysis of results of DSBs induced by x‐ray from two methods and the heat map of the correlation analysis. * p < 0.05, ** p < 0.01, *** p < 0.001, **** p < 0.0001; for each group, n = 3. Figure S7: The standard curve method results of model organisms samples treated by H2O2. (A) mice. (B) Arabidopsis thaliana . (C) Saccharomyces cerevisiae . (D) Escherichia coli . Figure S8: Detection of DSBs in mice whole blood induced by H2O2. (A) The neutral SCGE results of mice samples treated by H2O2. (B) Analysis of results of DSBs induced by H2O2 from standard curve method and neutral SCGE and the heat map of the correlation analysis. [file JCLA-39-e70123-s001.docx]

Supplementary Figures


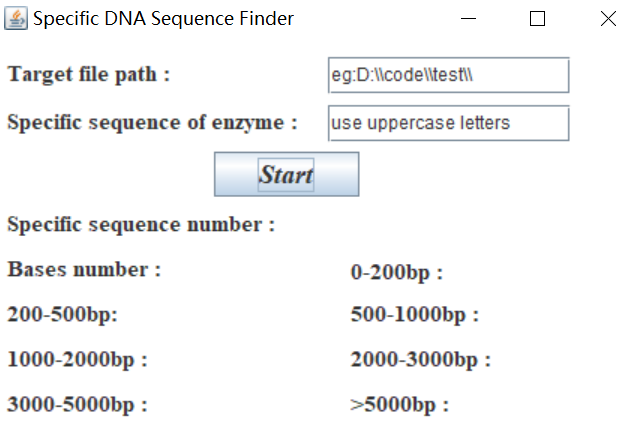

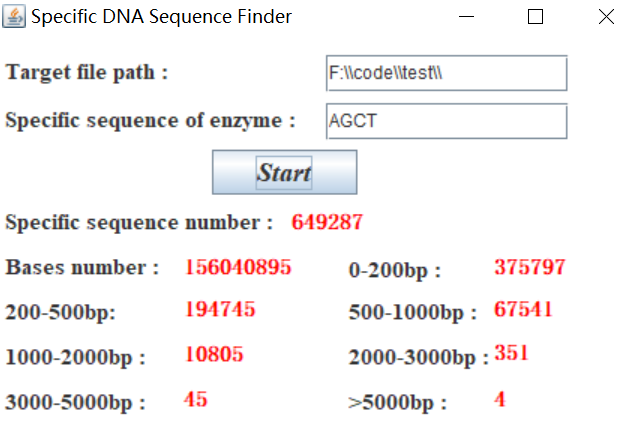


A

B

**Figure S1.** The interface figure of the software “Specific DNA Sequence Finder”. (A) The software interface figure. (B)The identification result of restriction enzyme AluI on human X chromosome DNA by software.


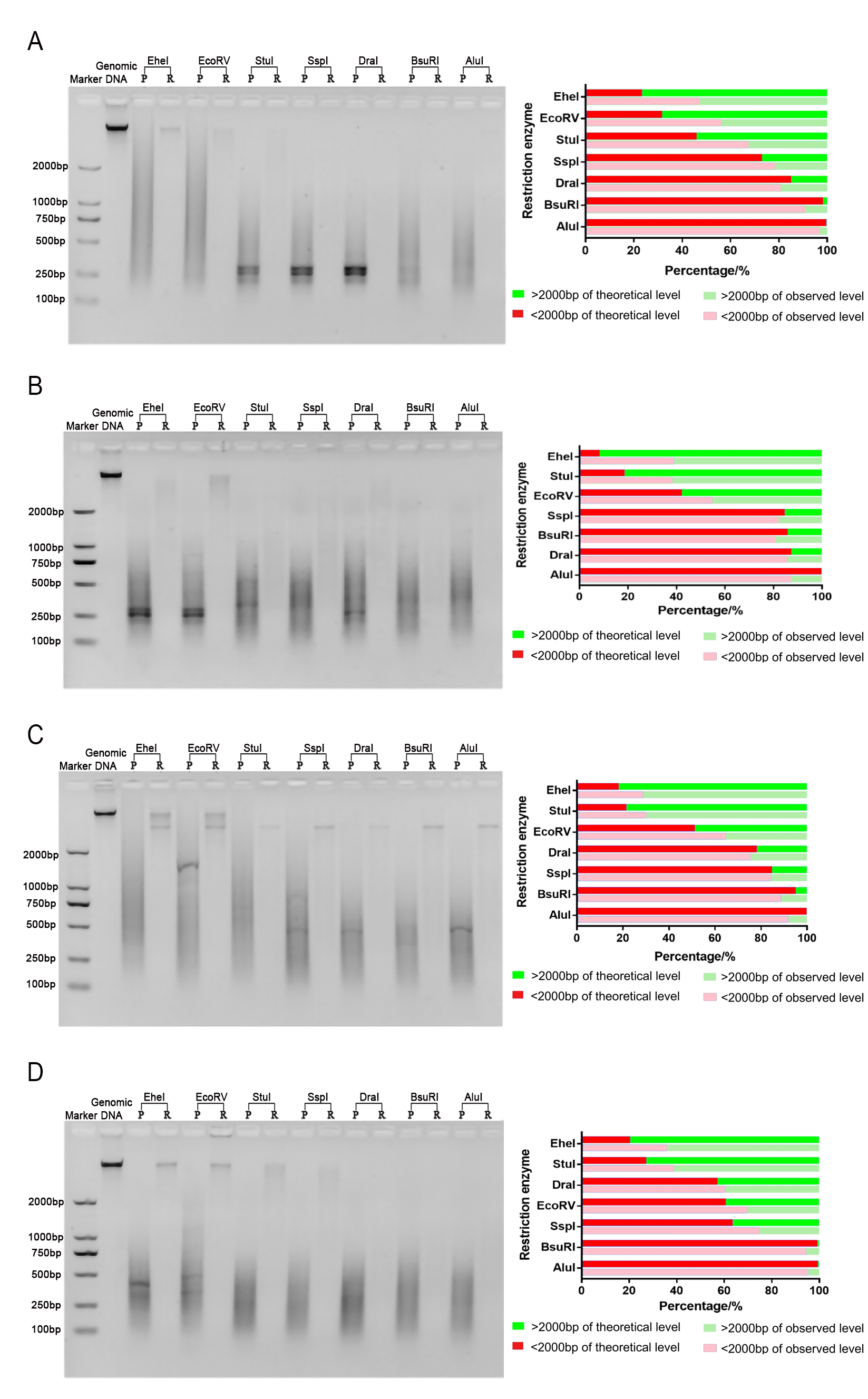


**Figure S2.**The results of agarose gel electrophoresis and gray value analysis of enzyme digestion extraction products in model organisms. (A) mice. (B) *Arabidopsis thaliana*. (C) *Saccharomyces cerevisiae*. (D) *Escherichia coli*. P: the LM-qPCR amplification products; R: the enzyme digestion products.

**Figure S3.**The LM-qPCR amplification curves and the Ct-lgNDSBs (different DNA size fragements) standard curves of model organisms standards. (A) mice. (B) *Arabidopsis thaliana*. (C) *Saccharomyces cerevisiae*. (D) *Escherichia coli*.


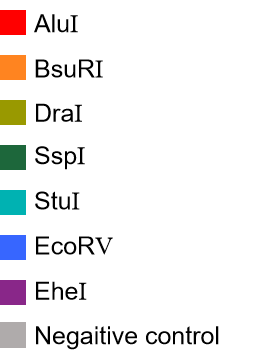

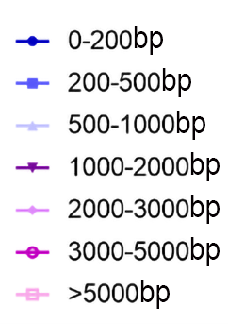

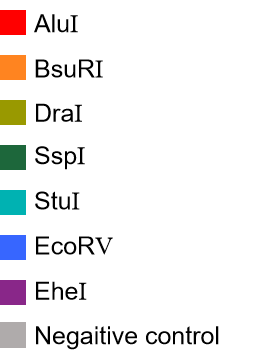

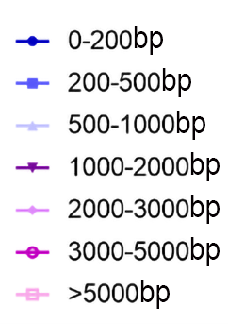

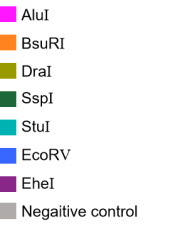

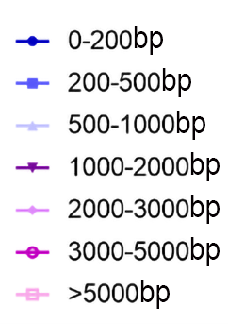

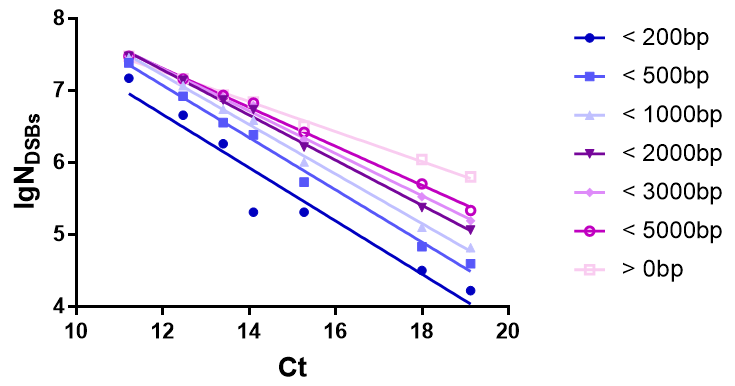

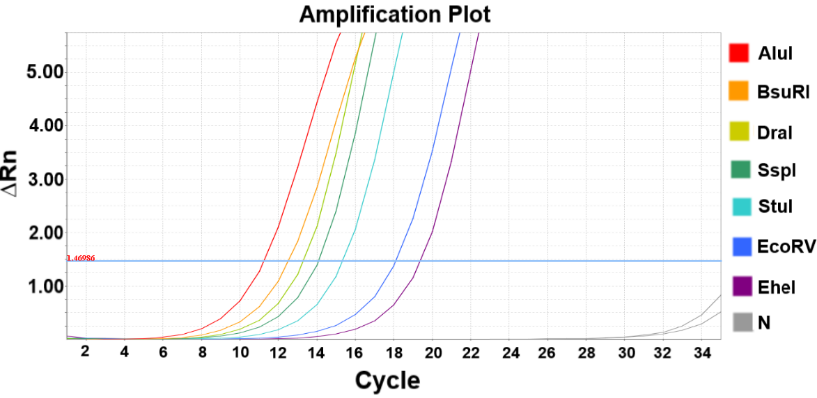

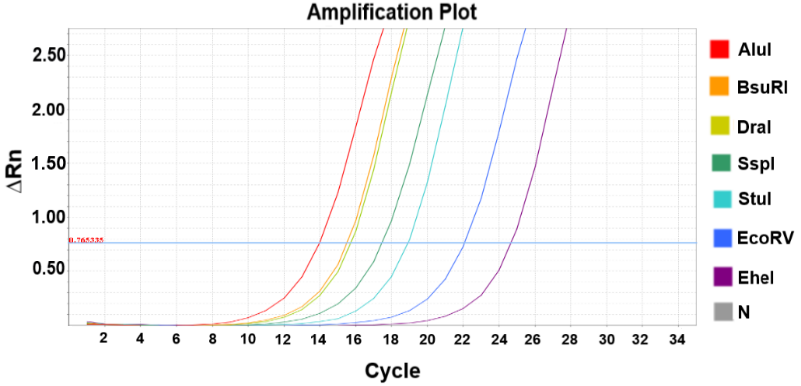

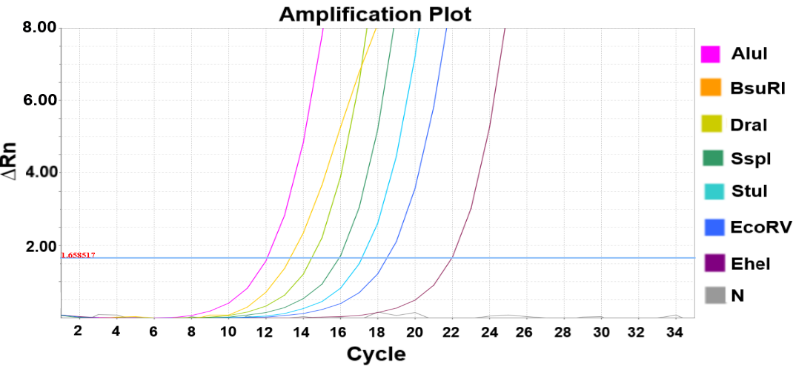

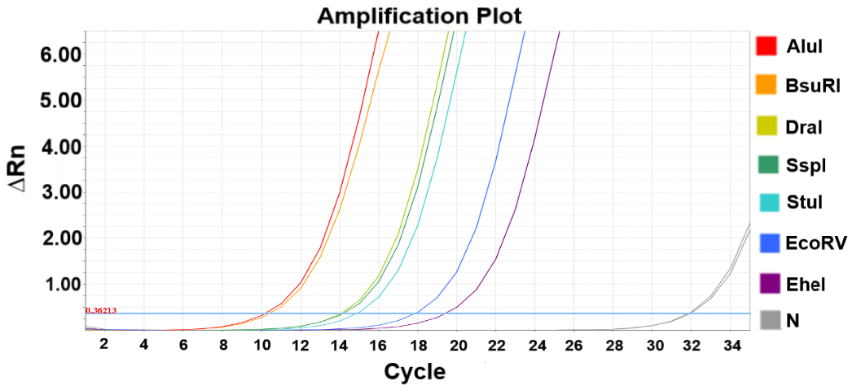

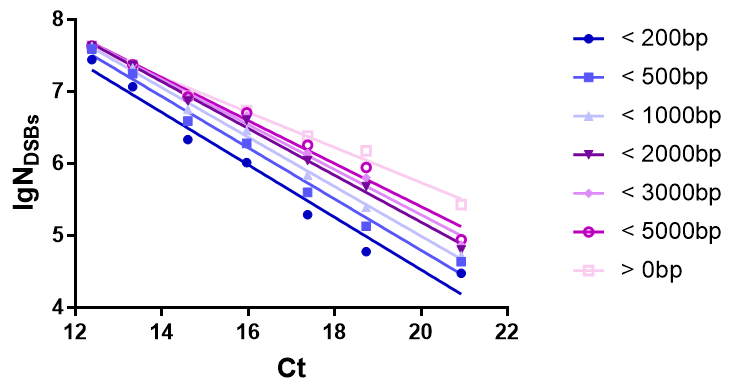

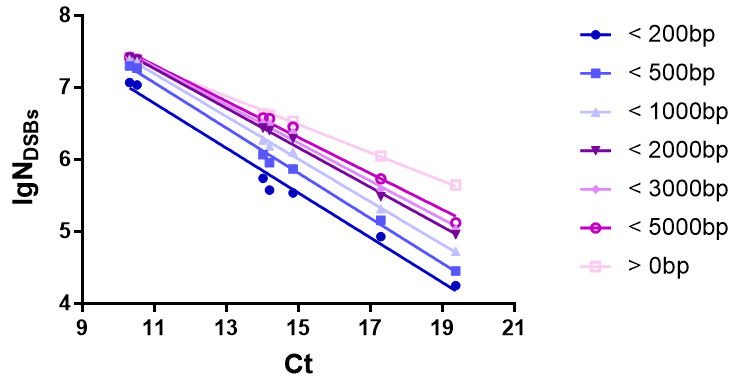

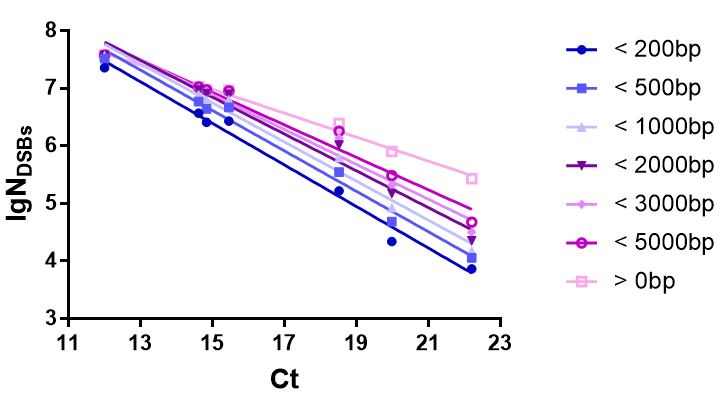


C

D


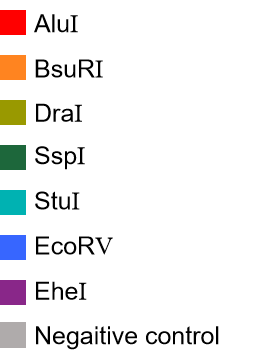

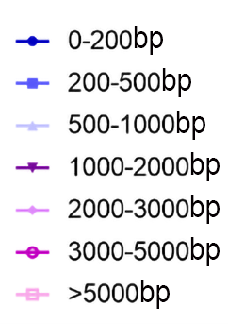


A

B


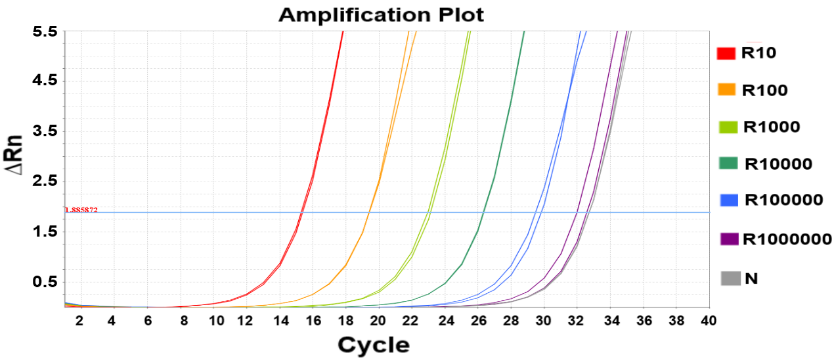

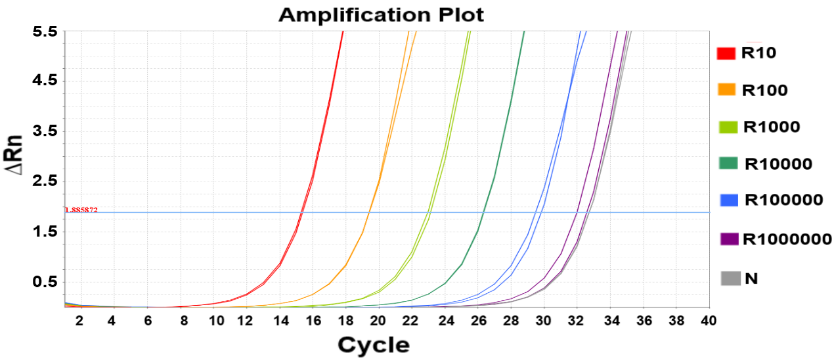
**Figure S4.** The LM-qPCR amplification curves and the fitting curves after 10-10^6^ times dilution of model organisms standards. (A) mice. (B) *Arabidopsis thaliana*. (C) *Saccharomyces cerevisiae*. (D) *Escherichia coli*.

A


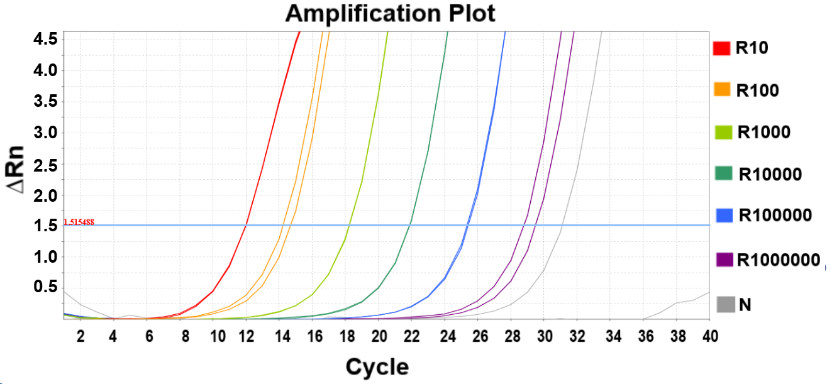

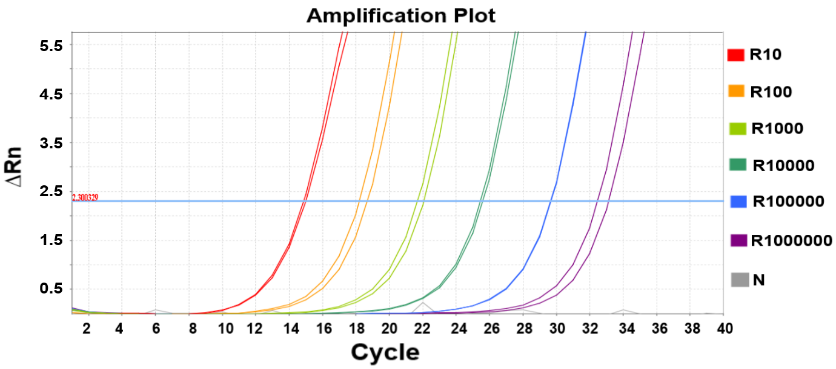

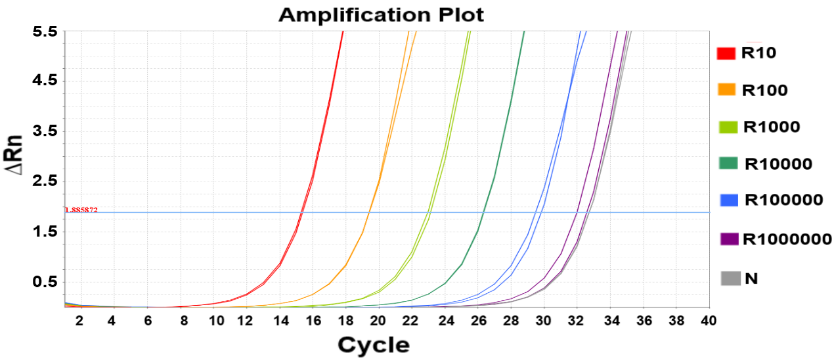


C

D

B


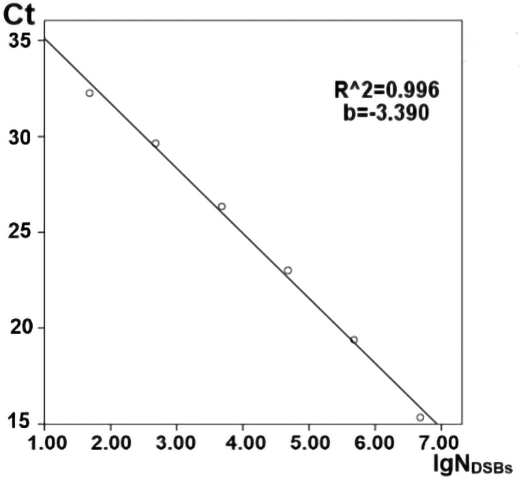

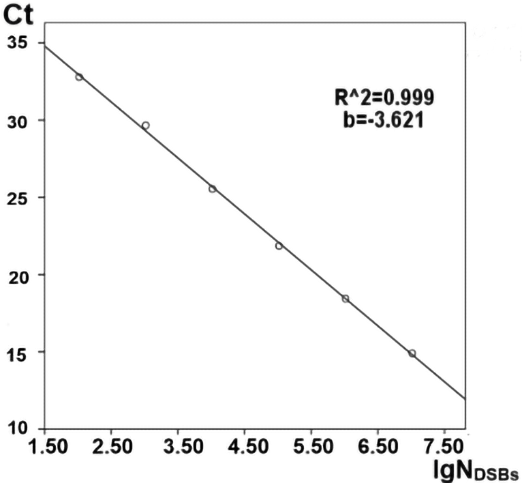

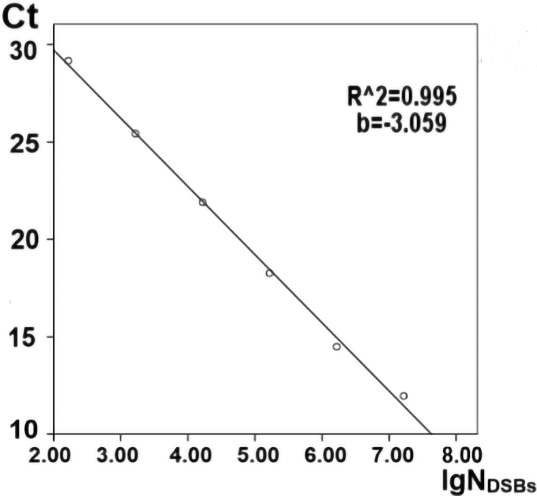

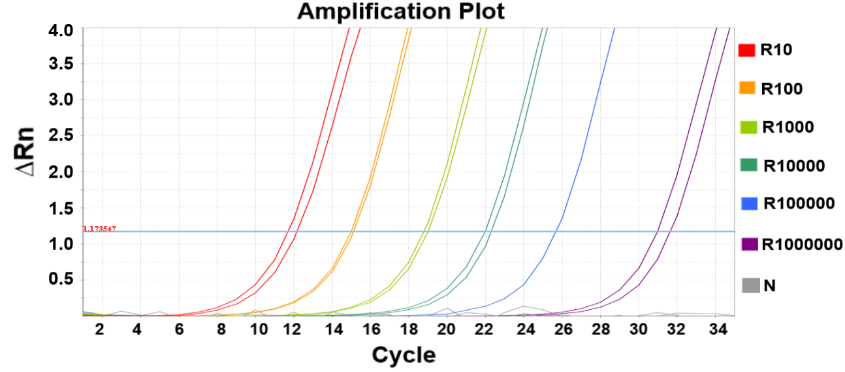

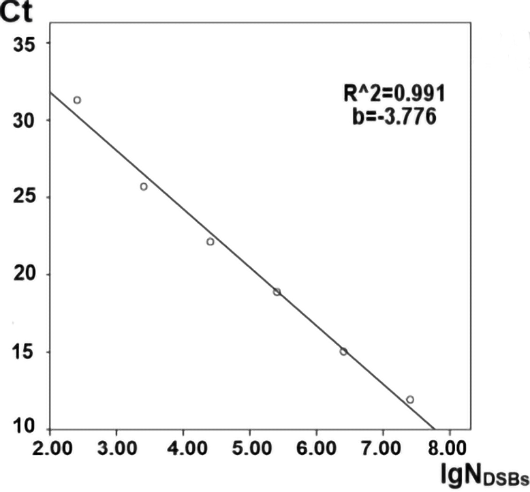

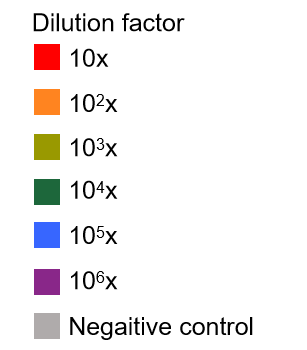

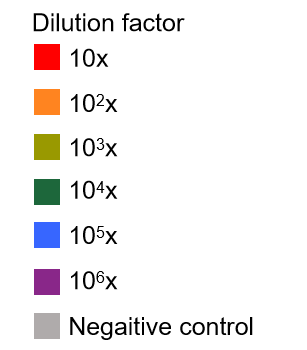

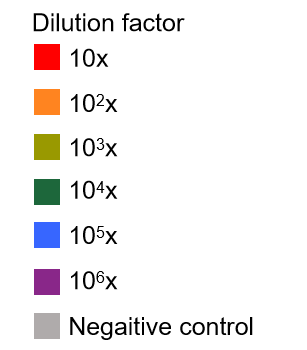

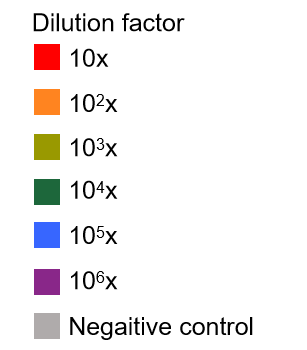


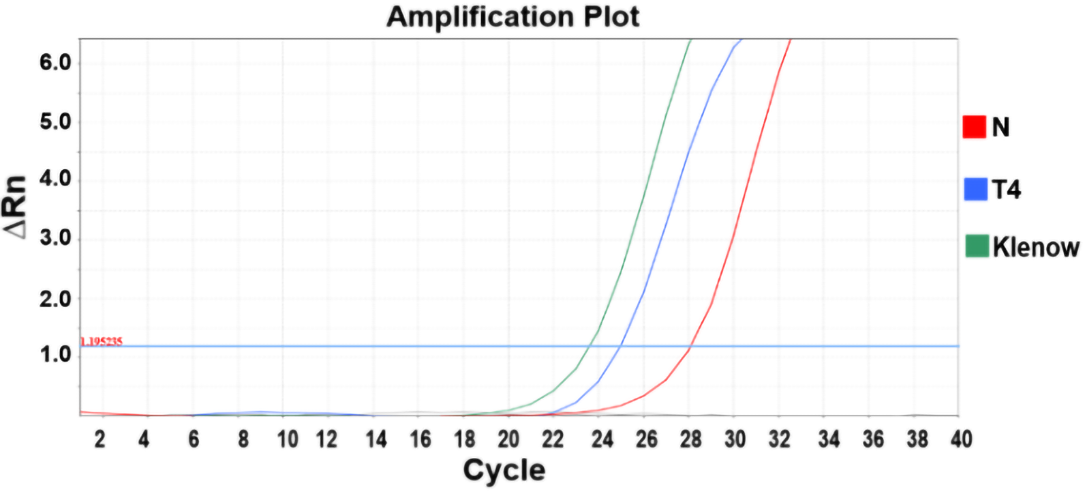


**Figure S5.** LM-qPCR amplification curves of using Klenow fragment, T4 DNA polymerase or not to flatten the sticky ends.


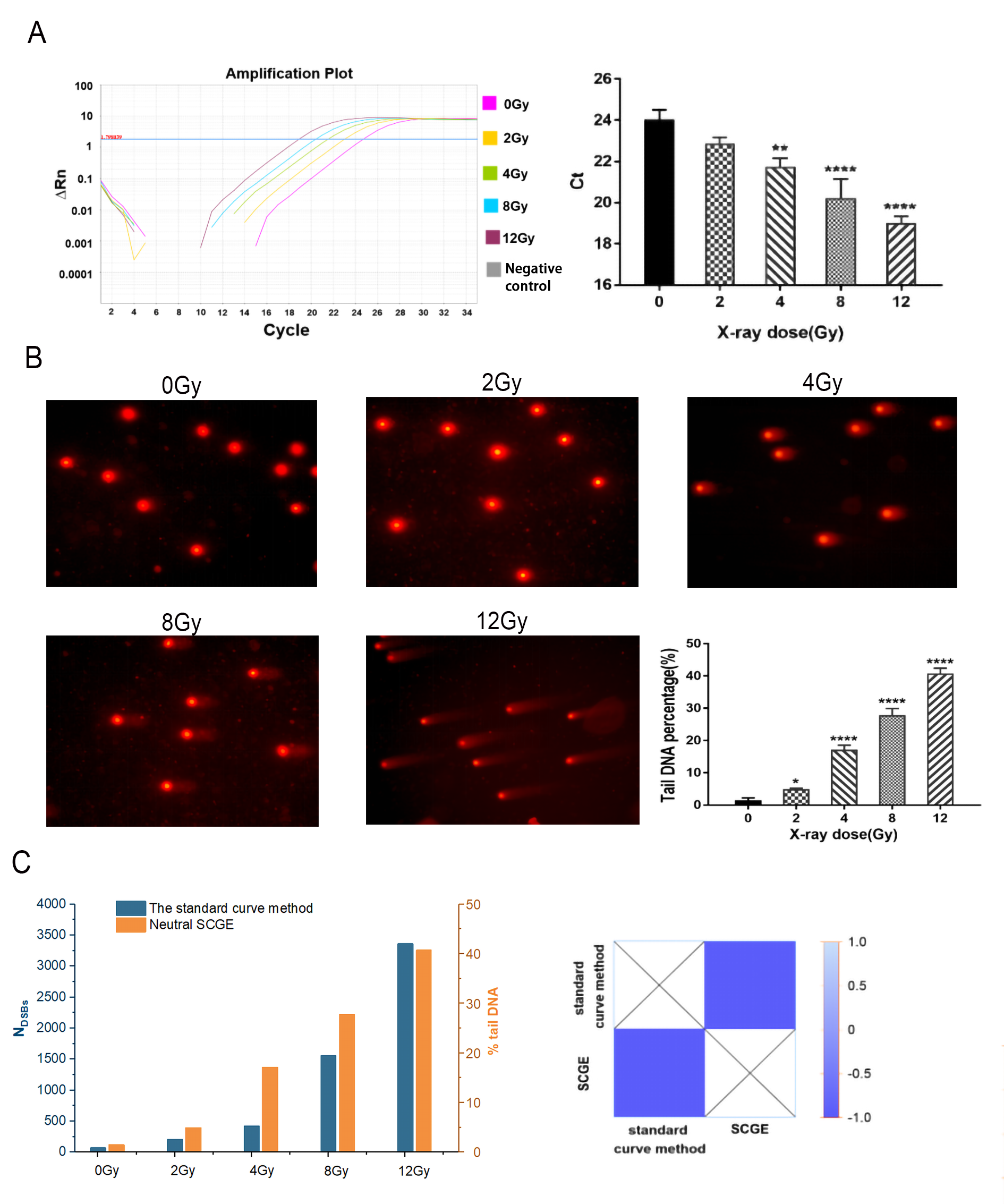
**Figure S6.** Detection of DSBs in mice whole blood induced by X-ray using the standard curve method and neutral SCGE. (A) The standard curve method results of DSBs induced by X-ray. (B) Neutral SCGE results of DSBs induced by X-ray. (C) Analysis of results of DSBs induced by X-ray from two methods and the heat map of the correlation analysis. * *P* < 0.05, ** *P* < 0.01, *** *P* < 0.001, **** *P* < 0.0001; for each group, n = 3.

**Figure S7.** The standard curve method results of model organisms samples treated by H_2_O_2_. (A) mice. (B) *Arabidopsis thaliana*. (C) *Saccharomyces cerevisiae*. (D) *Escherichia coli*.

C


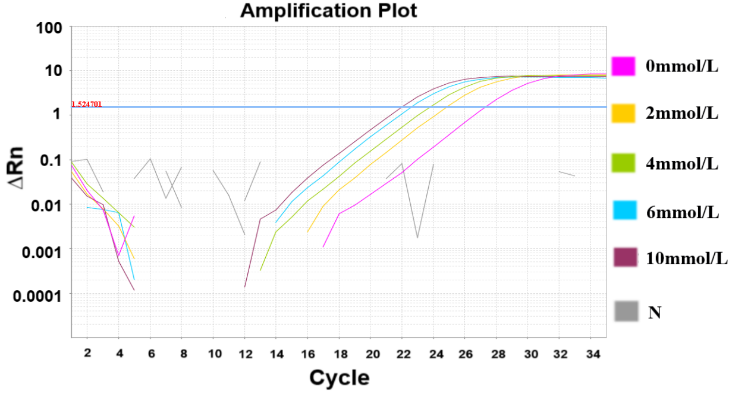

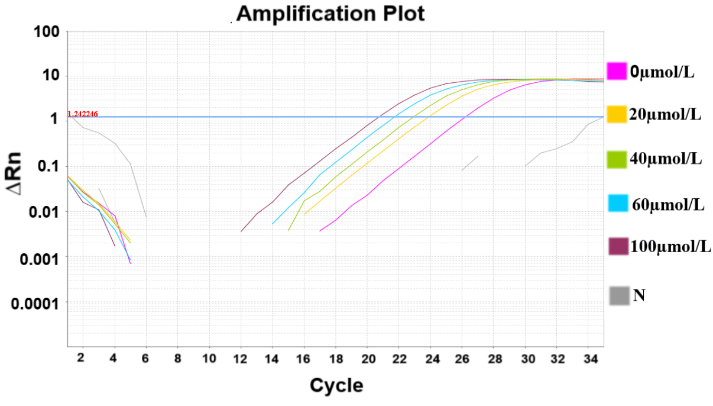


B

A


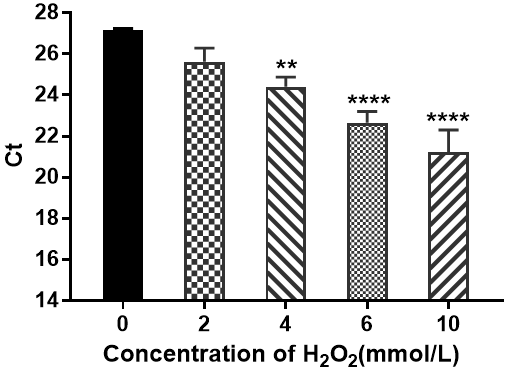

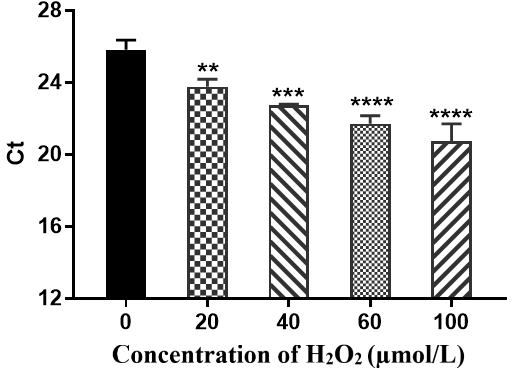

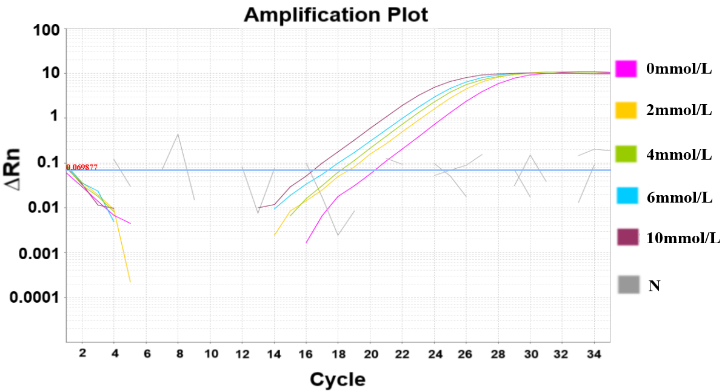

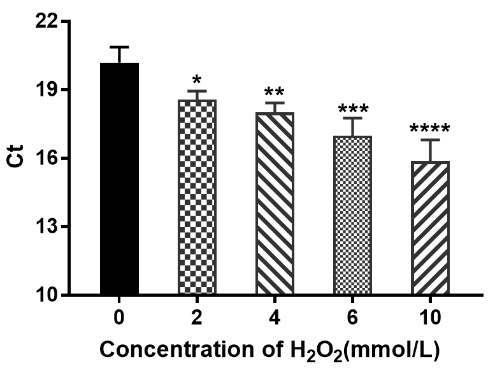

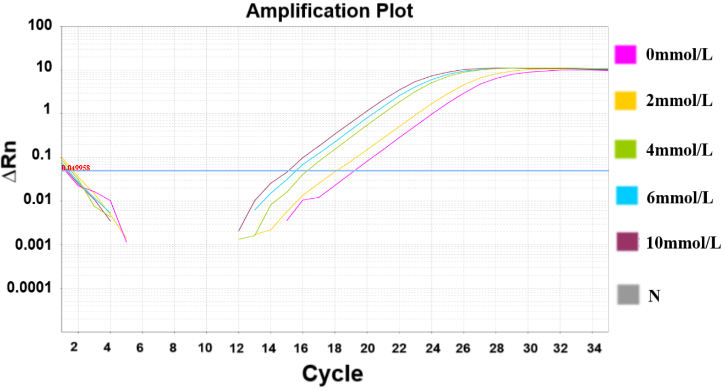

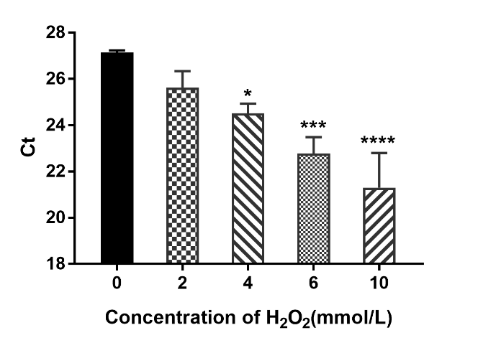


D


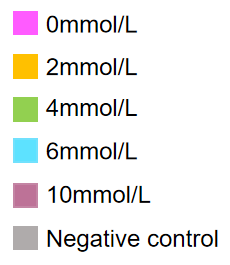

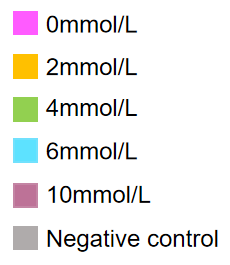

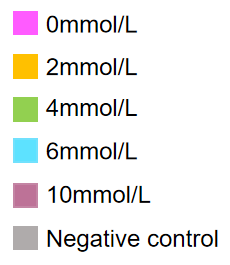

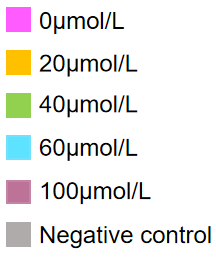

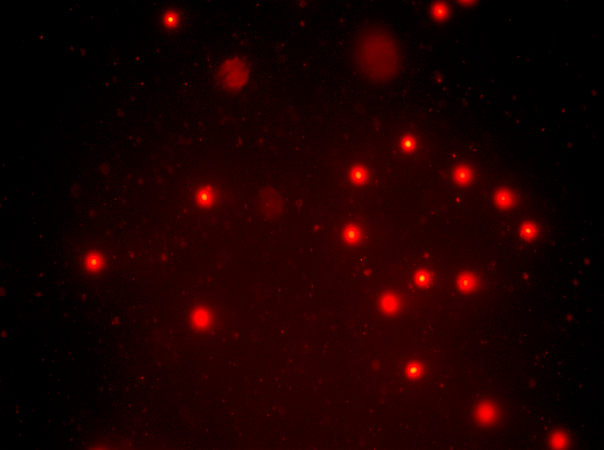

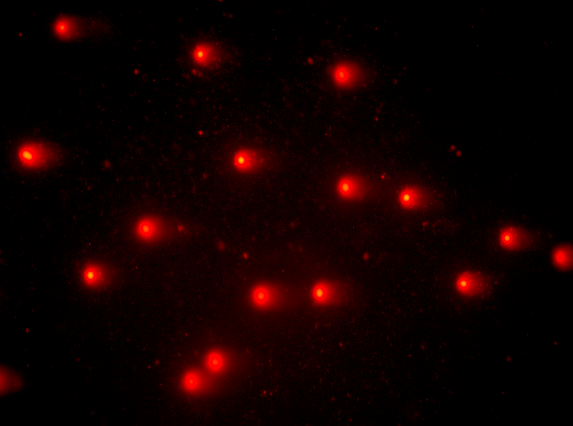

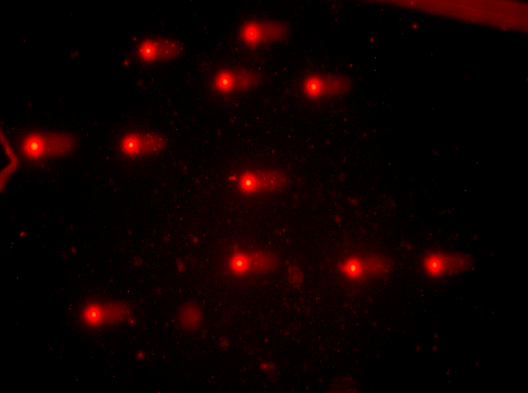

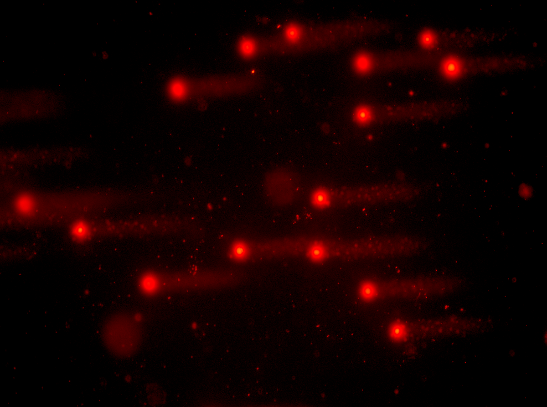


0µmol/L 20µmol/L 40µmol/L

60µmol/L 100µmol/L **100µmol/L**


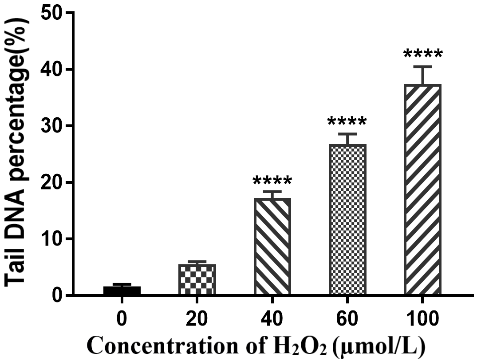

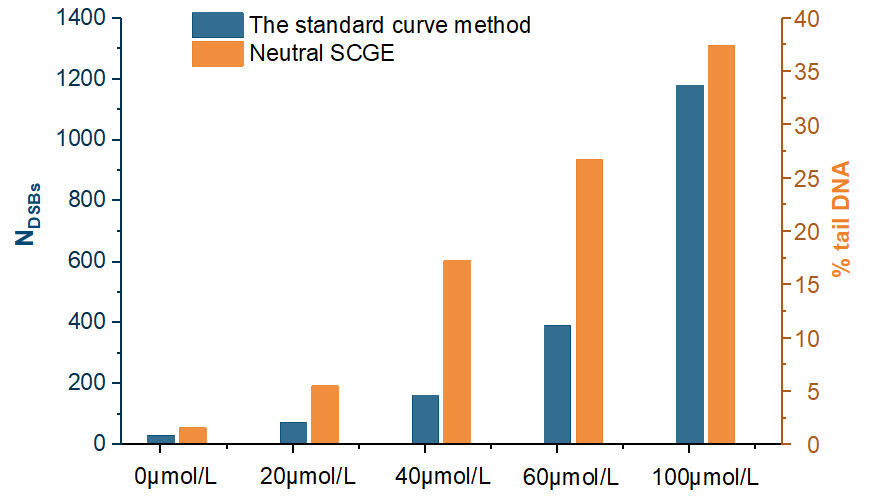

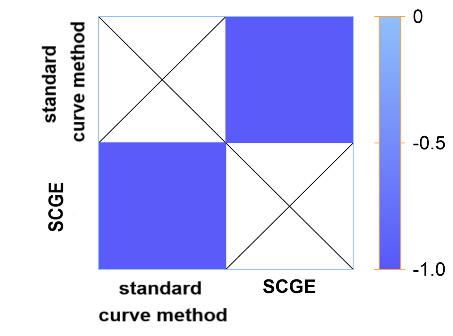

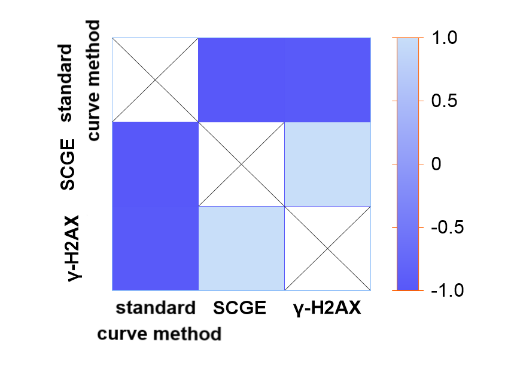


A

B


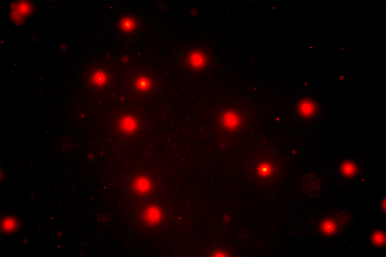


**Figure S8.** Detection of DSBs in mice whole blood induced by H_2_O_2_. (A) The neutral SCGE results of mice samples treated by H_2_O_2_. (B) Analysis of results of DSBs induced by H_2_O_2_ from standard curve method and neutral SCGE and the heat map of the correlation analysis.
