# Supplementary material for: Quantitative Analysis of DNA Double‐Strand Breaks in Genomic DNA Using Standard Curve Method
Source: J Clin Lab Anal. 2025 Oct 18;39(23):e70123. doi: 10.1002/jcla.70123 (PMC12699195; doi:10.1002/jcla.70123)
Supplement: Supplementary file 2 — Table S1: The recognized sequence of seven blunt‐terminal restriction endonucleases. Table S2:. The reaction condition of LM‐qPCR. Table S3: The results of human PBMCs treated with x‐ray. Table S4: The results of human PBMCs treated with H2O2. Table S5: The results of mice sample treated with x‐ray. Table S6: The results of mice sample treated with H2O2. [file JCLA-39-e70123-s002.docx]

### Supplementary Tables

**Table S1.** The recognized sequence of seven blunt-terminal restriction endonucleases

| Restriction endonuclease | Recognized sequence | Restriction endonuclease | Identification sequence | |
| --- | --- | --- | --- | --- |
| AluI | 5’-AGCT-3’ | StuI | 5’-AGGCCT-3’ | |
| BsuRI | 5’-GGCC-3’ | EcoRV | 5’-GATATC-3’ | |
| DraI | 5’-TTTAAA-3’ | EheI | 5’-GGCGCC-3’ | |
| SspI | 5’-AATATT-3’ |  |  |  |

**Table S2**. The reaction condition of LM-qPCR

| Step | Temperature (^。^C) | Time (s) |
| --- | --- | --- |
| 12bp oligonucleotide release | 72 | 420 |
| Pre degeneration | 98 | 120 |
| Degeneration | 98 | 10 |
| Annealing | 68 | 10 |
| Extension | 68 | 120 |
| (35 cycles) | | |
| Melting curve analysis | 95 | 15 |
|  | 60 | 60 |
| Collect the melting curve from 60 °C to 90 °C | | |

**Table S3.** The results of human PBMCs treated with X-ray

| Detection method | The dose of X-ray (Gy) | | | | |
| --- | --- | --- | --- | --- | --- |
|  | 0 | 2 | 4 | 8 | 12 |
| The standard curve method（N_DSBs_） | 1752.18±  106.59 | 4463.99±  1106.85 | 8059.33±  2160.55 | 21331.22±  14522.73 | 40506.22±  22203.37 |
| Neutral SCGE (%Tail DNA) | 2.78±  1.37 | 12.08±  3.29 | 20.61±  1.44 | 34.04±  5.05 | 46.37±  2.25 |
| γ-H2AX technology  (average fluorescence intensity) | 288.70±  3.61 | 778.90±  4.60 | 1188.00±10.83 | 2467.00±  12.84 | 3567.00±  12.36 |

**Table S4.** The results of human PBMCs treated with H_2_O_2_

| Detection method | The concentration of H_2_O_2_ (µmol/L) | | | | |
| --- | --- | --- | --- | --- | --- |
|  | 0 | 20 | 40 | 60 | 100 |
| The standard curve method（N_DSBs_） | 717.73±  228.19 | 894.25±  154.27 | 1432.35±265.77 | 2817.65±  673.43 | 5961.19±  2243.06 |
| Neutral SCGE (%Tail DNA) | 2.50±  1.39 | 11.97±  2.23 | 20.49±  0.56 | 29.55±  2.14 | 44.87±  3.45 |
| γ-H2AX technology  (average fluorescence intensity) | 218.40±  6.36 | 407.70±  12.81 | 578.90±  16.55 | 1132.00±  41.07 | 1401.00±  54.12 |

**Table S5.** The results of mice sample treated with X-ray

| Detection method | The dose of X-ray (Gy) | | | | |
| --- | --- | --- | --- | --- | --- |
|  | 0 | 2 | 4 | 8 | 12 |
| The standard curve method（N_DSBs_） | 74.19±  27.51 | 206.22±  77.53 | 425.31±  202.87 | 1562.50±  1063.26 | 3365.58±  858.31 |
| Neutral SCGE (%Tail DNA) | 1.54±  0.68 | 4.94±  0.29 | 17.17±  1.37 | 27.84±  2.09 | 40.76±  1.65 |

**Table S6.** The results of mice sample treated with H_2_O_2_

| Detection method | The concentration of H_2_O_2_ (µmol/L) | | | | |
| --- | --- | --- | --- | --- | --- |
|  | 0 | 20 | 40 | 60 | 100 |
| The standard curve method（N_DSBs_） | 31.01±  22.47 | 73.45±  15.43 | 160.81±  45.69 | 391.06±  195.58 | 1178.53±  472.47 |
| Neutral SCGE (%Tail DNA) | 1.59±  0.40 | 5.52±  0.52 | 17.27±  1.13 | 26.76±  1.86 | 37.48±  3.05 |
